# Tubulin polyglutamylases TTLL4C and TTLL6B are essential for maintaining cytoskeletal integrity in *Trypanosoma brucei*

**DOI:** 10.1128/msphere.00909-25

**Published:** 2026-04-30

**Authors:** Lucas Brehm, Moritz Röder, Stephanie Lamer, Marinus Thein, Andreas Schlosser, Carlo Unverzagt, Klaus Ersfeld

**Affiliations:** 1 Molecular Parasitology, Department of Biology, University of Bayreuth 98920, Bayreuth, Germany; 2 Bioorganic Chemistry, Department of Chemistry, University of Bayreuth 160460, Bayreuth, Germany; 3 Rudolf Virchow Center, Center for Integrative and Translational Bioimaging, Julius-Maximilians-University Würzburg, Würzburg, Germany; Cleveland State University, Cleveland, Ohio, USA

**Keywords:** *Trypanosoma*, cytoskeleton, cell biology, post-translational modifications, microtubules

## Abstract

Tubulin polyglutamylation is a post-translational modification that modulates microtubule interactions with associated proteins and motor proteins, thereby contributing to the regulation of microtubule dynamics. Although its roles are well established in higher eukaryotes, the functional significance of this modification in protozoan parasites remains poorly understood. In the present study, we characterize two polyglutamylases, TTLL4C and TTLL6B, in the parasite *Trypanosoma brucei*, a protozoan organism that possesses a subpellicular, nematic array of highly stable microtubules. Using gene knockout and overexpression approaches combined with immunofluorescence, western blotting, and mass spectrometry, we show that TTLL4C functions as an initiator for α-tubulin polyglutamylation, specifically catalyzing monoglutamylation at residue E445. Loss of TTLL4C perturbs posterior cytoskeletal architecture, resulting in blunt cell ends and reduced cell length. By contrast, TTLL6B functions as an elongase with preferential activity on β-tubulin, extending polyglutamate chains after initiation. TTLL6B depletion results in an elongated cell morphology, organelle mispositioning, and delayed cytokinesis. Together, these findings delineate complementary roles of TTLL4C and TTLL6B in maintaining cytoskeletal integrity and cell shape in *T. brucei*, underscoring the importance of balanced polyglutamylation for morphogenesis and cell cycle progression in kinetoplastids.

**IMPORTANCE:**

Post-translational modifications of microtubules, collectively known as the tubulin code, are increasingly recognized as key determinants in the modulation of microtubule properties. Notably, these modifications have been implicated in the pathogenesis of several diseases, including specific forms of neurodegeneration and ciliopathies. A comprehensive understanding of this regulatory layer is therefore of considerable biological and medical significance. Moreover, the conservation of tubulin post-translational modifications across eukaryotic evolution underscores their fundamental cellular importance. In this study, the protozoan parasite *Trypanosoma brucei* is used as a model system to examine the functional roles of two microtubule polyglutamylases, TTLL4C and TTLL6B. The findings reveal that these enzymes are essential for maintaining cytoskeletal integrity, cell morphology, organelle positioning, and normal cell growth. Collectively, this work advances our understanding of microtubule regulation and highlights the broader cellular functions governed by tubulin polyglutamylation.

## INTRODUCTION

Microtubules are cylindrical polymers composed of α- and β-tubulin heterodimer subunits (1). In eukaryotic cells, they fulfill a wide range of essential functions, including organelle organization, intracellular transport, and cellular motility, when organized as axonemes in cilia and flagella. They are also indispensable for chromosome segregation. In many protozoan species, microtubules are also essential for the definition of cell shape or the organization of specific structures, such as the conoid in apicomplexa or the ventral disc in *Giardia* (2–4). This expanded microtubule functionality is particularly evident in kinetoplastids, where a nematic array of subpellicular microtubules gives these parasites their spindle-like morphology (5).

In contrast to the highly dynamic cytoplasmic microtubule cytoskeleton often observed in higher eukaryotes, the cytoskeleton of kinetoplastids is remarkably stable (6). Nevertheless, morphological changes that occur during the parasites’ life cycle, in conjunction with the cytoskeletal remodeling during cell division, necessitate a certain degree of microtubule dynamic behavior (7–9). The regulation of stability and the requirement for periodic microtubule remodeling are poorly understood in kinetoplastids. Despite the identification of numerous cytoskeleton-associated proteins (MAPs), the specific contribution of these proteins to cytoskeletal organization remains to be fully elucidated. Furthermore, kinetoplastids have evolved a unique set of such proteins with little or no similarity to known microtubule-associated proteins in other eukaryotes (5, 6).

A notable exception to this low degree of evolutionary conservation is the presence of enzymes that catalyze a range of microtubule post-translational modifications (PTMs) (10, 11). The best characterized tubulin modifications are acetylation, polyglycylation, polyglutamylation, and detyrosination/tyrosination (11). Acetylation takes place on a single lysine residue (K40) located within the microtubule lumen, whereas polyglutamylation, polyglycylation, and detyrosination occur on the unstructured and exposed C-terminal tails of both α- and β-tubulin. In kinetoplastids, acetylation, polyglutamylation, and the detyrosination/tyrosination cycle are present, while polyglycylation is absent (12–14).

Polyglutamylation is defined as the process of adding one or more glutamate residues to the primary amino acid chain of both α- and β-tubulin. The addition of the first branching glutamate residue is formed via an isopeptide bond between a glutamate of the primary amino acid chain and a newly added glutamate residue, with further chain elongation achieved through conventional peptide bond formation. Several glutamic acid residues within the C-terminal tails of α- and β-tubulin can be modified in this manner. Polyglutamylation is catalyzed by a family of enzymes known as tubulin-tyrosine-like ligases (TTLLs), which are named for the sequence homology of their respective catalytic domains with those of the founding member of this enzyme family, the tubulin-tyrosine ligase (TTL) (15, 16). Interestingly, the TTL enzyme does not function as a glutamylase but rather catalyzes the re-addition of tyrosine to the C-terminus of α-tubulin and, in kinetoplastids, to β-tubulin as well (12).

The presence of multiple TTLL enzymes within a single organism is indicative of their catalytic specialization. Some act as initiases, adding the first branching glutamate and thus forming the initial isopeptide bond. In contrast, others serve as elongases, with the capacity to extend the chain to up to 15 glutamic acid residues (17). It is reasonable to hypothesize that, given the presence of two or three potential modification sites on the tubulin tails, distinct TTLLs exhibit site-specific initiation activities (18).

Based on sequence homology, eight members of the TTLL family have been identified in the parasite *Trypanosoma brucei* (TTLL1, 4A, 4B, 4C, 6A, 6B, 9, 12B). All of these are assigned to the polyglutamylase subgroup (10). No polyglycylases, which also belong to the TTLL family, have been identified, and no biochemical evidence for polyglycylation has been found (12).

In some model systems, several M14-type cytosolic carboxypeptidases (CCPs) have been described to counteract TTLL activities and function as tubulin deglutamylases (19–21). Despite the identification of CCP homologs in *T. brucei* (22), their function in tubulin-deglutamylation remains to be elucidated.

Significant progress has been made in understanding the function of tubulin polyglutamylation in model systems of higher eukaryotes, ranging from cultured cells to transgenic mice (11). At a molecular level, it has been shown that this PTM can fine-tune the interaction between specific microtubule-associated proteins and motor proteins with microtubules (23–25). At a physiological level, perturbations in polyglutamylation can lead to defects, such as impaired flagella-mediated cell motility, male infertility, and neurodegeneration (26). However, the role of this modification in protozoa remains less well understood. In previous studies, we have shown that the depletion of individual TTLLs (TTLL1, 6A, and 12B) in *Trypanosoma brucei* via RNA interference or gene deletion results in severe defects in cytoskeletal organization and aberrant motility patterns (27, 28). In the present study, gene deletion is utilized to investigate the role of the *T. brucei* polyglutamylases TTLL4C and TTLL6B. Using a novel monoclonal antibody that specifically recognizes monoglutamylated α-tubulin, we demonstrate that TTLL4C functions as an α-tubulin-specific initiator. Cells devoid of TTLL4C exhibit pronounced defects in their overall cell morphology, particularly with regard to the formation of the posterior cell pole. In contrast, TTLL6B preferentially elongates polyglutamate chains on β-tubulin. Cells depleted of TTLL6B also exhibit morphological abnormalities, characterized by an elongated phenotype distinct from that of TTLL4C-deficient cells. Furthermore, TTLL6B depletion impairs cell growth and disrupts cell cycle progression.

## RESULTS

### TTLL4C acts as an initiator for α-tubulin polyglutamylation

Building upon previous research on the *Trypanosoma brucei* polyglutamylases TTLL6A, TTLL12B (27), and TTLL1 (28), this study extends our analysis to include two additional members of the TTLL family, TTLL4C and TTLL6B (for TTLL nomenclature in *T. brucei*, see reference 10). These enzymes have not yet been characterized in *T. brucei*, and their *in vivo* activities remain unknown. In order to address this gap, targeted knockout and overexpression experiments were performed for both enzymes.

For TTLL4C, a gene knockout was successfully generated via homologous recombination using antibiotic resistance cassettes, replacing the entire open reading frames of both alleles (Fig. S1A). Following integration, real-time quantitative PCR (RT-qPCR) analysis confirmed the absence of detectable *TTLL4C* transcripts (Fig. S1B), validating the successful establishment of the knockout cell line.

In order to investigate the *in vitro* effects of TTLL4C depletion, specific antibodies targeting different glutamyl side chains on α- and β-tubulin were applied. While our previous studies utilized the GT335 antibody, which was raised against a bi-glutamylated octapeptide derived from α-tubulin (29) and detects glutamyl side chains of any length on both trypanosomal tubulins (28), we sought to refine the analysis with more specific antibodies. Therefore, an antibody specific for monoglutamylated α-tubulin at residue E445 was generated and was termed αMonoE. This antibody was produced using a γ1-monoglutamylated α-tubulin peptide as the immunogen (Fig. S1C). Its specificity was validated through an antibody-binding competition assay. In this assay, the αMonoE antibody displayed exclusive reactivity toward trypanosomal cytoskeletons when preincubated with the linear peptide. Conversely, preincubation with the monoglutamylated peptide abolished binding, thus confirming its specificity for the monoglutamylated modification (Fig. S1D). Using immunofluorescence to investigate the localization of the αMonoE signal, a non-uniform signal pattern was observed in wild-type cells (Fig. S2A). The posterior end was more intensely labeled than the anterior parts of the cell body. Interestingly, this antibody also strongly stained the mitotic spindle (Fig. S2A). The entire flagellum is labeled, whereas the growing, new flagellum was more intensely stained than the mature flagellum (Fig. S2B). In addition, the PolyE antibody was used to detect glutamyl side chains longer than three residues on α- or β-tubulin, and the βMonoE antibody (30) was employed to identify monoglutamylated β-tubulin at residue E435 (31).

**Fig 1:**
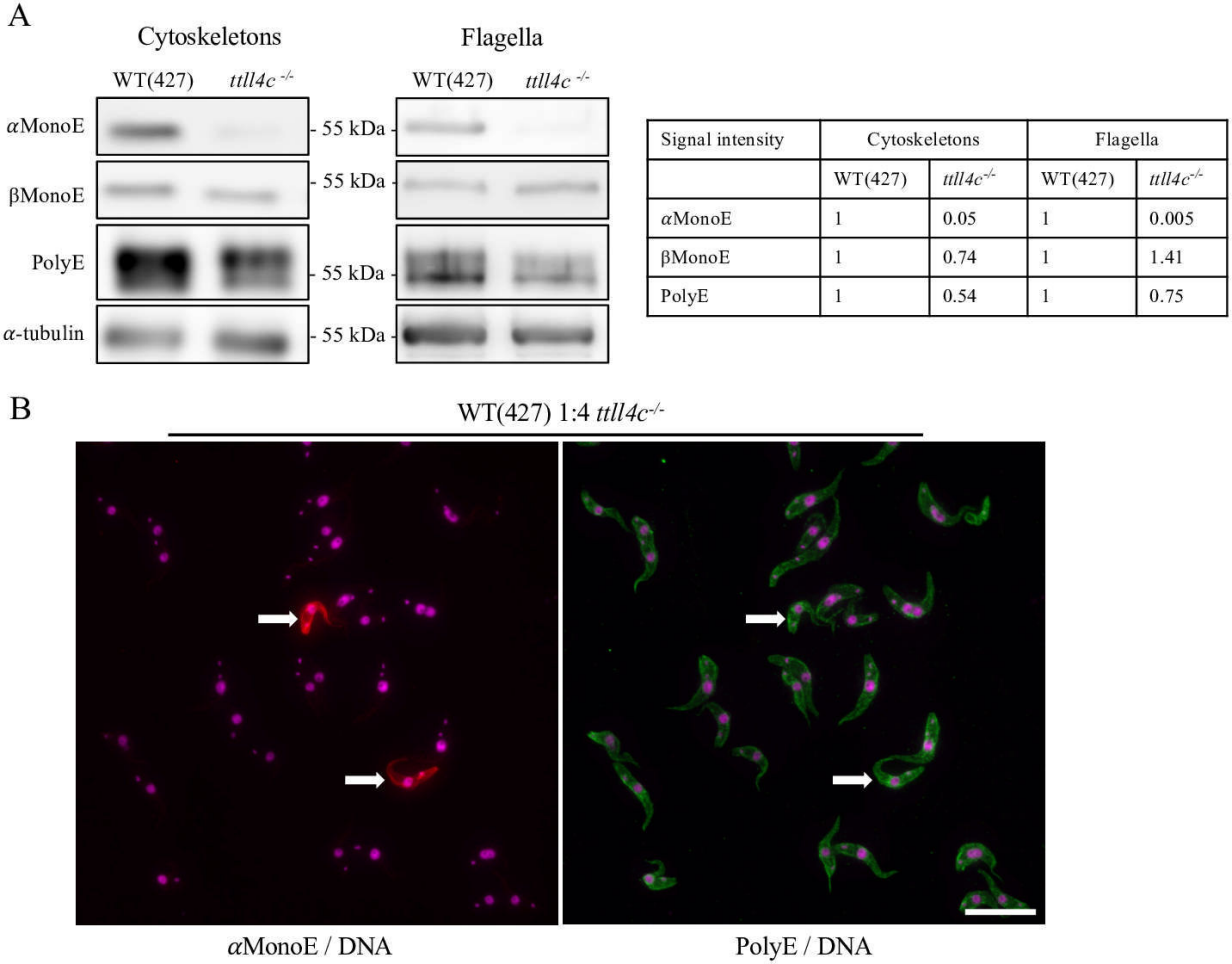
Reduced microtubule glutamylation following TTLL4C depletion. (**A**) Western blot analysis of cytoskeletal and flagellar fractions derived from wild-type (WT, strain 427) and *ttll4c^−/−^* mutant cells. The αMonoE antibody detects monoglutamylation of α-tubulin at residue E445, while βMonoE recognizes monoglutamylation of β-tubulin at residue E435. PolyE antibody identifies polyglutamylation with chains longer than three residues on either α- or β-tubulin (upper band: α-tubulin, lower band: β-tubulin). α-tubulin (TAT1) serves as a loading control. Data shown are representative of three independent experiments. The table on the right presents quantification of three independent western blot results, normalized to α-tubulin levels in WT samples. (**B**) Immunofluorescence microscopy of WT and *ttll4c^−/−^* cells. WT and mutant cells were mixed at a 1:4 ratio and labeled with αMonoE (red) and PolyE (green) antibodies. DNA (nucleus and kinetoplast) was stained using DAPI (magenta). Wild-type cells are marked with arrows. Images are representative of three independent experiments. Scale bar: 20 µm.

Using these antibodies, a comparative analysis was conducted on cytoskeletal and flagellar preparations from *ttll4c^−/−^* cells and those of the parental wild-type cell line. Western blot analysis demonstrated an almost complete disappearance of the αMonoE signal in both the cytoskeletal and flagellar fractions, indicating loss of monoglutamylation of α-tubulin E445. In contrast, the PolyE signal exhibited only a moderate reduction for α-tubulin in the cytoskeletal fraction (~46%), suggesting a limited impact on longer glutamyl side chains occurring on other sites with the tubulin tails. Notably, the βMonoE signal remained unaltered in *ttll4c^−/−^* cells, indicating that the monoglutamylation of β-tubulin E435 was unaffected by the knockout (Fig. 1A). These findings are consistent with mass spectrometry data obtained from isolated cytoskeletons, which revealed that the knockout of TTLL4C results in the presence of non-glutamylated α-tubulin within both the tyrosinated and detyrosinated tubulin pools (“0 Glu peak” in Fig. S3). In order to further investigate the impact of TTLL4C knockout on polyglutamylation, immunofluorescence microscopy was used to analyze wild-type and *ttll4c^−/−^* cells, mixed in a 1:4 ratio, using the αMonoE and PolyE antibodies. While αMonoE staining was absent in both *ttll4c^−/−^* cells and their flagella, the PolyE signal was not visibly altered (Fig. 1B).

The almost complete loss of the αMonoE signal indicates that TTLL4C possesses initiase activity, which is responsible for adding the first glutamic acid to the side chain of α-tubulin E445. Consequently, depletion of TTLL4C should lead to a reduction in the PolyE signal, as fewer branching points are available for elongation by the corresponding elongase. This can indeed be observed for the upper band on the western blot probed with PolyE antibody (Fig. 1A). However, the signal is not entirely absent. This can be explained if one of the other seven identified polyglutamylases (10) partially compensates for the loss of TTLL4C, which may also account for the weak αMonoE signal observed in *ttll4c^−/−^* cytoskeletons.

### TTLL4C-mediated polyglutamylation is integral to the proper organization of the posterior microtubule cytoskeleton

The subpellicular cytoskeleton of *Trypanosoma brucei* is a highly organized array of microtubules located beneath the plasma membrane, contributing to the parasite’s unique morphology and structural stability. This cytoskeletal array, which defines the helical shape of the cell, consists of approximately 100 microtubules in the central region that progressively decrease in number toward the anterior and posterior ends. At the posterior end, the microtubules form a tapered, open-pipe-like structure with a diameter of approximately 0.5–1.0 µm (8, 32). The integrity of the microtubule array depends on the dynamic regulation of microtubules by various MAPs and PTMs (5).

**Fig 2:**
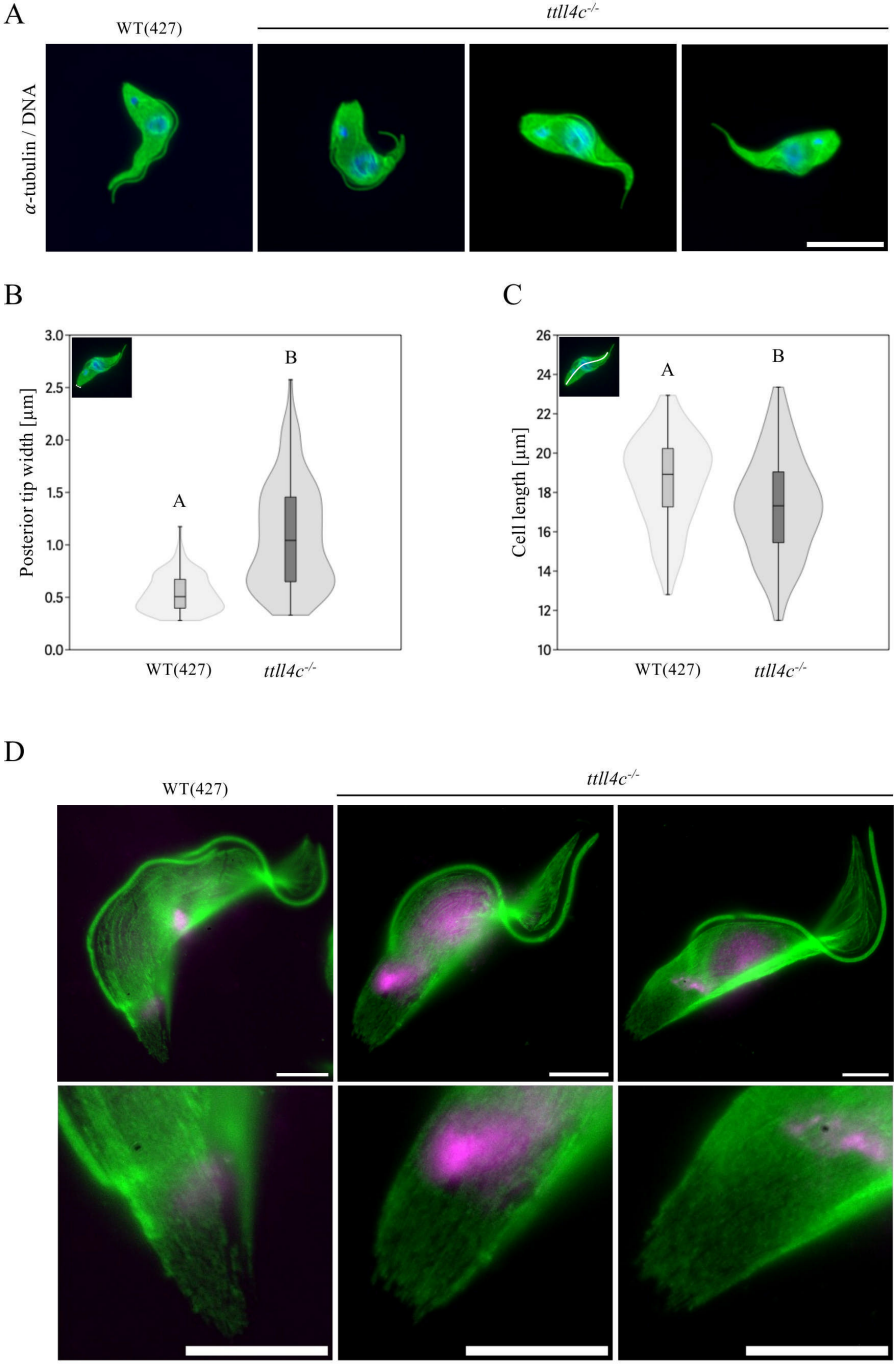
Morphological characterization of TTLL4C-deficient cells. (**A**) Immunofluorescence microscopy of wild-type (WT) and *ttll4c^−/−^* cells. Microtubules were visualized using an α-tubulin antibody (TAT1, green), while DNA was labeled with DAPI (blue). Scale bar: 10 µm. (**B**) Analysis of posterior tip width distribution in G1-phase WT and *ttll4c^−/−^* mutant cells. Tip width was measured as indicated in the upper left corner. (**C**) Violin plots depicting the distribution of cell lengths in WT and *ttll4c^−/−^* mutant cells. Cell lengths of G1-phase cells were measured from the posterior to the anterior tip through the approximate center of the cell (insert upper left corner). The plots display the arithmetic mean, interquartile range, standard error, and density curve of the data points. Sample size (*N*) = 100 cells. Statistical significance between groups is denoted by different letters, with identical letters indicating a lack of statistical significance (Mann-Whitney pairwise test, *P* < 0.05). The data presented are representative of three independent experiments. (**D**) Expansion microscopy of WT and *ttll4c^−/−^* cytoskeletons. Microtubules were stained with an α-tubulin antibody (TAT1, green) and DNA was visualized with DAPI (magenta). Upper panels show whole-cell views; lower panels display magnified images of the posterior tip. For better visualization, expansion microscopy images were processed using the ImageJ plugin “Sharpen.” Scale bars: 20 µm.

As previously described for the *ttll1^−/−^* cell line (28), *ttll4c^−/−^* cells exhibited a disrupted posterior microtubule array (Fig. 2A). Their posterior ends appeared blunt, with an average diameter of 1.08 µm—double that of the wild type (0.54 µm)—and a maximum aperture of ~2.6 µm (Fig. 2B). Furthermore, depletion of TTLL4C resulted in a reduction in cell length, with knockout cells measuring 17.3 µm compared to 18.6 µm in wild-type cells (Fig. 2C). We utilized expansion microscopy to investigate the cytoskeletal ultrastructure in detail. In wild-type cells, microtubules converge to form a pointed structure at the posterior cell pole. In contrast, *ttll4c^−/−^* cells exhibit a deficiency in this convergence, resulting in microtubules that uniformly terminate in a blunt end (Fig. 2D; Fig. S4). These observations suggest that the reduction in α-tubulin polyglutamylation may impair microtubule bundling at the posterior end.

**Fig 3:**
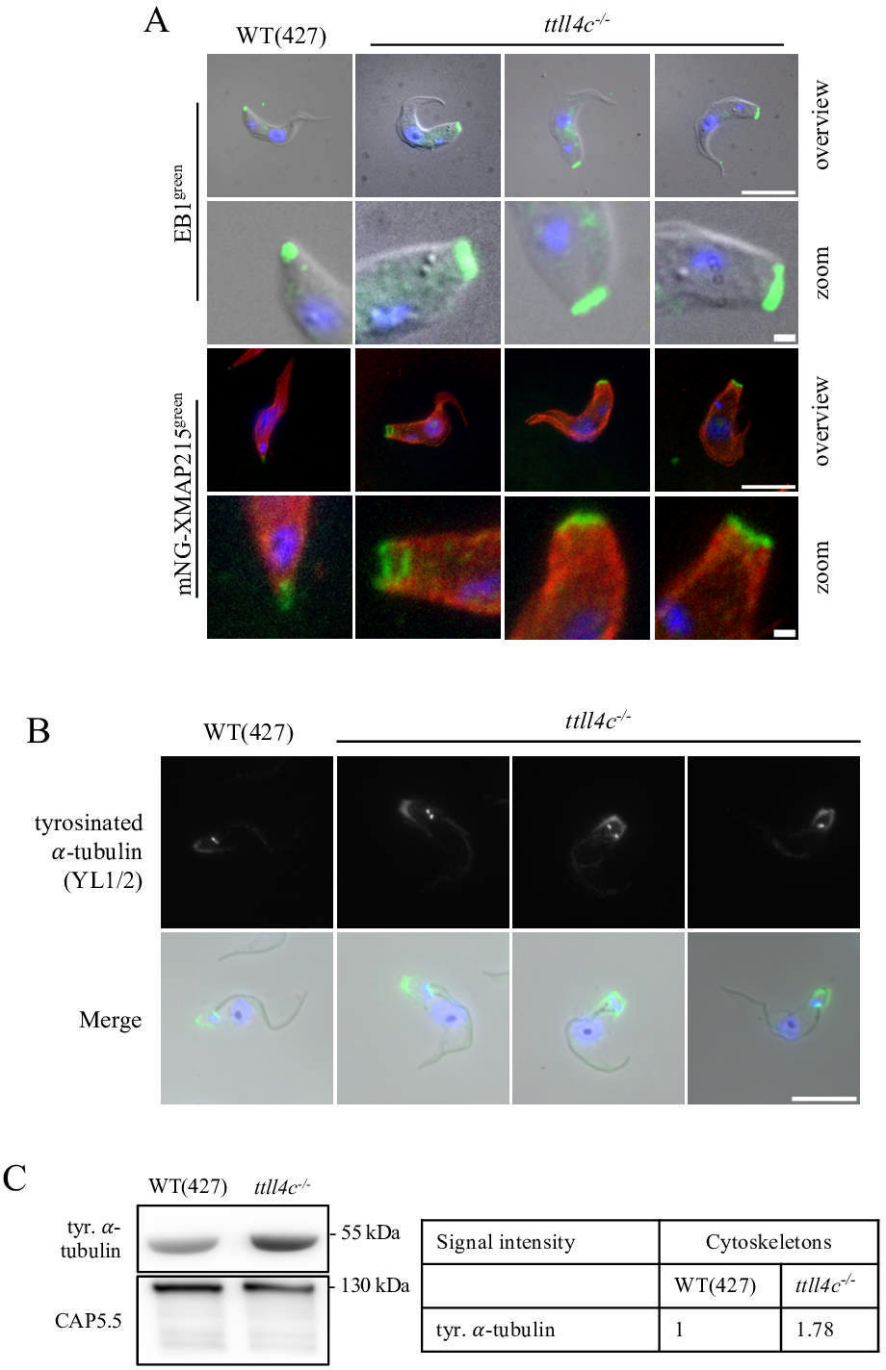
Impact of TTLL4C depletion on MAP binding and tubulin tyrosination. (**A**) Immunofluorescence microscopy of WT and *ttll4c^−/−^* cells. Upper panels: whole cells labeled with anti-EB1 antibody (green). Lower panels: cytoskeletons of WT and *ttll4c^−/−^* cells expressing endogenously tagged 3×Ty-mNeonGreen-3×Ty-XMAP215, stained with anti-Ty BB2 (green) and α-tubulin (TAT1, red) antibodies. DNA was visualized with DAPI (blue). The bottom rows provide a magnified view of the posterior cell tips. All images are representative of at least three independent experiments. Scale bars: 10 µm (whole cell view), 1 µm (zoomed images). (**B**) Immunofluorescence analysis of cytoskeletons from wild-type (WT) and *ttll4c^−/−^* cells. Upper panels: detection of tyrosinated α-tubulin using the YL1/2 antibody. Lower panels: merged images displaying DAPI-stained DNA (blue), tyrosinated tubulin (green), and phase contrast microscopy (gray). Scale bar: 10 µm. (**C**) Western blot analysis of cytoskeletal fractions from WT and *ttll4c^−/−^* cells, probed with cytoskeleton-associated protein (CAP5.5; loading control) and YL1/2 antibodies. The adjacent table presents normalized mean intensity values from three independent experiments.

In order to investigate whether the microtubule plus-end-binding proteins EB1 and XMAP215 adapt to the structural changes at the blunt posterior end, we examined their distribution. An anti-EB1 antibody was employed, and an endogenously tagged XMAP215 construct with an N-terminal mNeonGreen was generated. Our observations revealed that both EB1 and XMAP215 remained associated with the microtubule plus ends (Fig. 3A) and adjusted to the expanded posterior aperture in knockout cells. However, the presence of these proteins does not preclude the possibility that the altered glutamylation pattern modulates the functionality of these proteins, as has been observed for XMAP215 in other model systems (see Discussion).

Previous studies, including our own, have demonstrated a direct connection between microtubule polyglutamylation and tubulin detyrosination (28, 33). To investigate this relationship further, we used the YL1/2 antibody to stain for tyrosinated α-tubulin (Fig. 3B; Fig. S5A). In wild-type cells, the posterior region displayed only faint staining, whereas *ttll4c^−/−^* cells exhibited a marked increase in tyrosinated microtubules. Quantification of western blots indicated an approximately 1.8-fold increase in tyrosinated microtubules in cytoskeletal extracts from the knockout cells (Fig. 3C). This increase is likely attributable to an observed cross-talk between tyrosination/detyrosination and polyglutamylation (34). Collectively, these findings demonstrate that the regulatory relationship between microtubule polyglutamylation and tubulin detyrosination observed *in vitro* also operates in the context of TTLL4C activity in *Trypanosoma brucei*.

In addition to the observed morphological abnormalities, we assessed the growth and cell cycle progression of *TTLL4C*-deficient cells. This analysis revealed only a slight impact on growth, with the doubling time increasing from 9.6 h in wild-type cells to 10 h in *ttll4c^−/−^* cells (Fig. S5B), and no significant changes in the distribution of cell cycle stages (Fig. S5C).

### Phenotype rescue by ectopic TTLL4C expression

**Fig 4:**
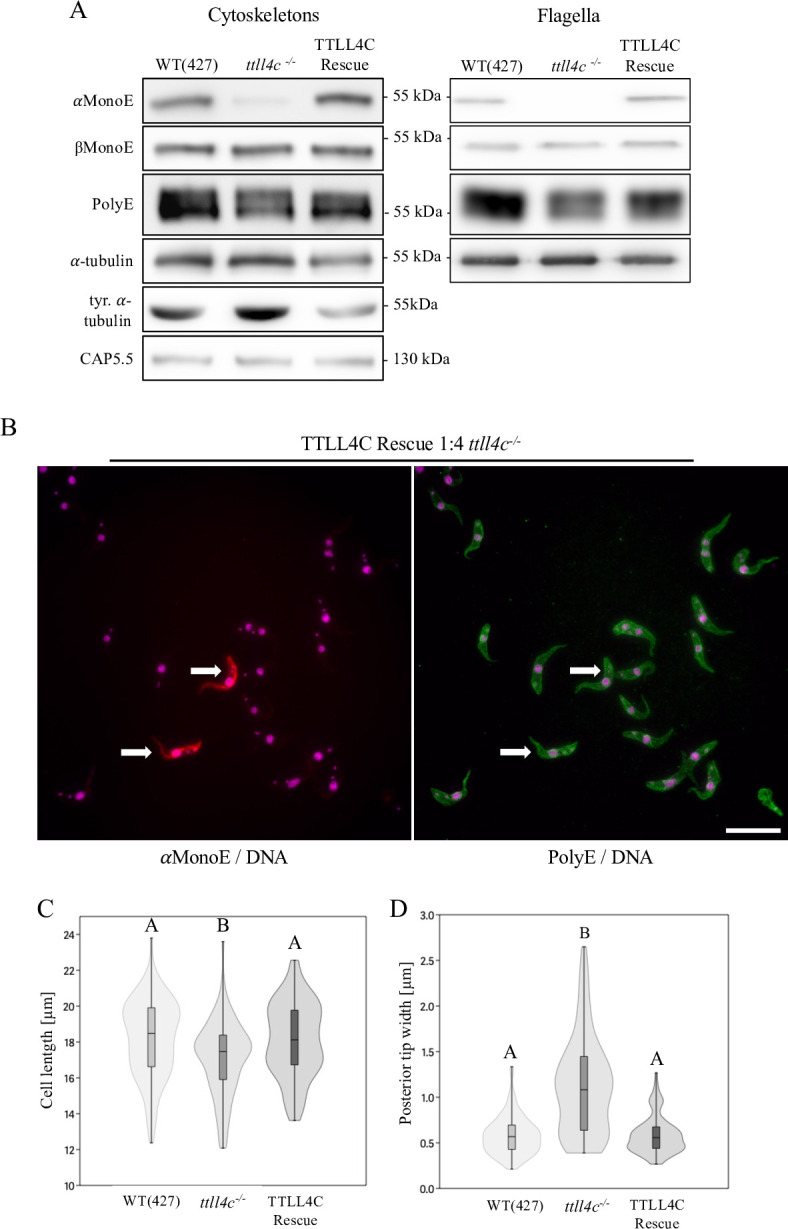
Restoration of WT phenotype upon TTLL4C expression. (**A**) Western blots of cytoskeletal and flagellar fractions of WT, *ttll4c*^−/−^, and TTLL4C rescue cells. α-tubulin (TAT1) and CAP5.5 were included as loading controls. The blots presented are representative of three independent experiments. (**B**) Immunofluorescence microscopy of TTLL4C rescue and *ttll4c^−/−^* cells. Rescue and mutant cells were mixed at a 1:4 ratio and stained with αMonoE (red) and PolyE (green) antibodies, while DNA (nucleus and kinetoplast) was stained with DAPI (magenta). Rescue cells are marked with arrows. Images are representative of three independent experiments. Scale bar: 20 µm. (**C**) Violin plots illustrating the distribution of cell lengths across WT, *ttll4c^−/−^*, and TTLL4C rescue cells. (**D**) Analysis of posterior tip width distribution in G1-phase cells from each respective cell line. The plots show the arithmetic mean, interquartile range, standard error, and density curve of the data. Sample size (*N*) = 100 cells. Statistical significance between groups is indicated by different letters; groups sharing identical letters are not statistically different (Mann-Whitney pairwise test, *P* < 0.05). The data presented are representative of three independent experiments.

In order to confirm the specificity of the TTLL4C deletion, two distinct constructs for ectopic TTLL4C expression were generated. Initially, we inserted the promoterless TTLL4C gene into the intergenic DNA of the tubulin gene array, utilizing polycistronic read-through transcription (Fig. 4) (35). The objective was to attain mRNA levels comparable to those measured in the wild type. RT-qPCR analysis of the *TTLL4C* transcript levels revealed that the rescue cell line exhibited approximately 60% of the wild-type expression level (Fig. S6A). Western blot analysis and immunofluorescence experiments demonstrated that αMonoE levels were restored to wild-type levels upon re-introduction of TTLL4C (Fig. 4A and B; Fig. S6B). Further morphological assessments confirmed that both the blunt posterior phenotype and cell shortening were rescued following the expression of an intact TTLL4C copy (Fig. 4C and D; Fig. S6C).

The second construct that was introduced was TTLL4C, tagged with 3xTy at the N-terminus and placed under the control of a strong, doxycycline-inducible promoter to achieve overexpression relative to wild-type cells (Fig. S7). RT-qPCR analysis showed an approximate 22-fold increase in TTLL4C expression relative to wild-type levels (Fig. S7A). However, this overexpression did not result in any noticeable alterations in glutamylation levels (Fig. S7B) or cellular phenotype (Fig. S7C through E). This absence of effect is not unexpected, given that TTLL4C acts as an initiator on α-tubulin, and the mass spectrometry data indicate that its substrate is nearly completely monoglutamylated in wild-type cells and hence saturated (Fig. S3).

Taken together, these findings demonstrate that TTLL4C is an initiator for α-tubulin polyglutamylation, specifically initiating monoglutamylation at residue E445. This was evidenced by a loss of the αMonoE signal and the accumulation of non-glutamylated α-tubulin in *Trypanosoma brucei ttll4c^−/−^* mutants. While TTLL4C deficiency caused distinct morphological defects, such as a disrupted posterior microtubule array and reduced cell length, its absence had only minor effects on growth, cytokinesis, and cell cycle progression. Furthermore, ectopic expression of TTLL4C partially restored these phenotypes, thereby underscoring its functional significance in maintaining microtubule organization.

### TTLL6B functions as a polyglutamate elongase with preferential activity on β-tubulin

**Fig 5:**
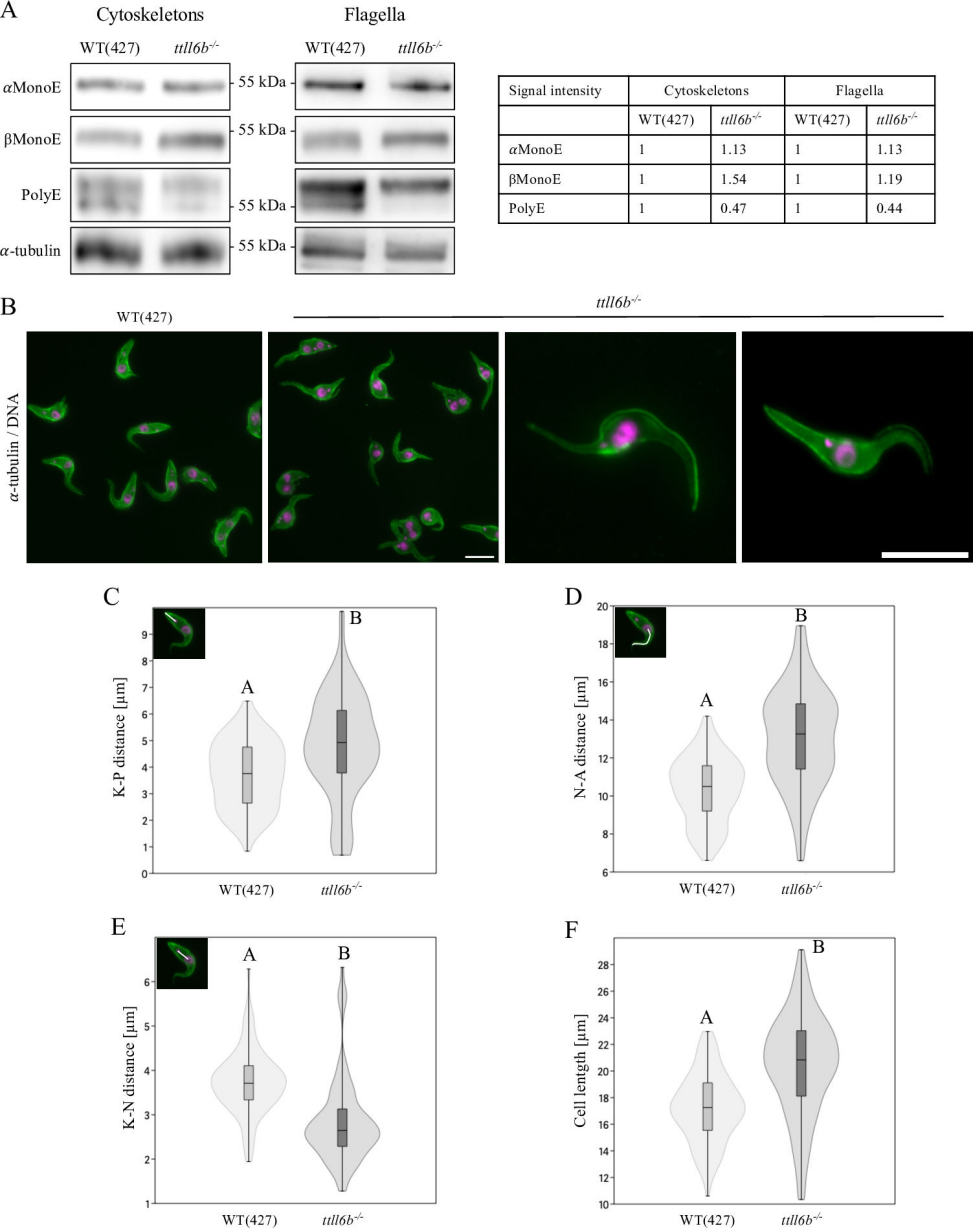
Characterization of TTLL6B-deficient cells. (**A**) Western blot analysis of cytoskeletal and flagellar fractions from wild-type (WT) and *ttll6b^−/−^* cells, with α-tubulin (TAT1) serving as a loading control. The accompanying table presents the quantification of three independent western blot results normalized to WT α-tubulin levels. (**B**) Immunofluorescence microscopy of wild-type (WT) and *ttll6b^−/−^* cells. Microtubules were labeled with an α-tubulin antibody (TAT1; green), and DNA was stained using DAPI (magenta). Scale bars: 10 µm. (**C**) Violin plots illustrating the distribution of the kinetoplast-to-posterior tip (K-P) length in WT and *ttll6b^−/−^* mutant cells. Posterior lengths of G1-phase cells were measured from the kinetoplast to the posterior tip along the approximate centerline of the cell (insert upper left corner). (**D**) Distribution of nucleus-to-anterior tip (N-A) length in G1-phase WT and *ttll6b^−/−^* cells, measured from the center of the nucleus to the anterior tip (upper left insert). (**E**) Analysis of the kinetoplast-to-nucleus (K-N) distance in G1-phase WT and *ttll6b^−/−^* cells. Distances were measured between the centers of the kinetoplast and nucleus, as indicated in the upper left insert. (**F**) Violin plots depicting the distribution of cell lengths in WT and *ttll6b^−/−^* mutant cells. Cell lengths of G1-phase cells were measured from the posterior to the anterior tip through the approximate center of the cell. The violin plots depict the arithmetic mean, interquartile range, standard error, and density curves of the measurements. Sample size (*N*) = 100 cells. Statistical significance between groups is indicated by letters, with different letters denoting statistical significance (Mann-Whitney pairwise test, *P* < 0.05). Data shown are representative of three independent experiments.

Following the characterization of TTLL4C, the investigation was expanded to TTLL6B, another putative tubulin polyglutamylase within the TTLL family (10). Using similar methodologies, we aimed to elucidate its enzymatic activity, substrate specificity, and distinctive effects on the cellular architecture, thereby advancing our understanding of tubulin modifications in *Trypanosoma brucei*. In accordance with the strategy that was employed for TTLL4C (Fig. S1A), a TTLL6B knockout cell line was generated, which was designated as *ttll6b^−/−^*. RT-qPCR analysis confirmed the complete absence of *TTLL6B* transcripts (Fig. S8A). In order to investigate changes in cytoskeletal and flagellar polyglutamylation, immunostaining of *ttll6b^−/−^* cells and probing cytoskeletal and flagellar extracts with antibodies specific to these modifications was performed (Fig. 5A; Fig. S8B and C). This analysis revealed a reduction in long polyglutamate chains (PolyE) to approximately 47% of wild-type levels in the trypanosomal cytoskeleton, with a more pronounced decrease in β-tubulin polyglutamylation. This was accompanied by an increase in monoglutamylated β-tubulin (βMonoE) to ~154% of wild-type levels and a slight increase in monoglutamylated α-tubulin (αMonoE) to ~113% of wild-type levels. Notably, the reduction in β-tubulin polyglutamylation was more evident in flagellar extracts than in cytoskeletal extracts (Fig. 5A). Furthermore, immunofluorescence images revealed that the reduction of long polyglutamate chains was primarily visible at the anterior and posterior regions of the microtubule array of the subpellicular cytoskeleton (Fig. S8B and C). This reduction was evident at non-saturating dilutions of the PolyE antibody (1:50,000 in contrast to 1:10,000; Fig. S9).

Based on these findings, it can be concluded that TTLL6B is not an initiator for the monoglutamylation of α-tubulin E445 and β-tubulin E435. Instead, the observed reduction in long polyglutamate chains (PolyE) in *ttll6b^−/−^* cells, coupled with the increase in monoglutamylated tubulin, provides strong evidence that TTLL6B functions as an elongase, responsible for extending polyglutamate chains after the initial monoglutamylation has occurred. Additionally, the more pronounced reduction in β-tubulin polyglutamylation compared to α-tubulin indicates that TTLL6B exhibits a preferential activity toward β-tubulin. The observed partial reduction in long glutamyl side chains is likely caused by the presence of additional elongases amongst the nine *T. brucei* TTLLs. For example, we previously characterized TTLL1 and identified this enzyme as an elongase (28).

### Depletion of TTLL6B affects the structure of the whole subpellicular cytoskeleton

Next, the phenotypic effects associated with the loss of TTLL6B by characterizing the *ttll6b^−/−^* cell line were examined. In contrast to the phenotype observed in *ttll4c^−/−^* cells, the TTLL6B knockout mutant exhibited an elongated shape, with a slender posterior and anterior end and a noticeably bulky nuclear region (Fig. 5B). The lengths of various cellular regions were measured in order to assess morphological changes in TTLL6B-deficient cells. Compared to wild-type cells (3.7 µm), TTLL6B-deficient cells exhibited a significantly increased kinetoplast-to-posterior distance, averaging 4.8 µm (Fig. 5C). Furthermore, the anterior portion of the cell was significantly elongated, with an average length of 13.1 µm, in contrast to 10.4 µm observed in the wild-type cells (Fig. 5D). Conversely, the distance between the kinetoplast and nucleus was reduced in the TTLL6B-deficient cells, decreasing from 3.7 µm in wild-type cells to 2.8 µm (Fig. 5E). These structural anomalies are concomitant with the diminished signal for polyglutamylation in the posterior and anterior portions of the subpellicular microtubule array of the knockout cells (Fig. S8B).

Next, we examined the levels of tyrosinated α-tubulin in *ttll6b^−/−^* cells. The loss of TTLL6B did not affect α-tubulin detyrosination (Fig. S10A), likely because the enzyme primarily elongates polyglutamate chains on β-tubulin, leaving α-tubulin, where the YL1/2 epitope resides, largely unchanged. As was also the case with *ttllc4*^−/−^ cells, the loss of TTLL6B did not affect the localization of EB1 (Fig. S10B).

In summary, the loss of TTLL6B in *ttll6b^−/−^* cells resulted in an elongated cell shape with altered cytoskeletal morphology and organelle mispositioning, including increased kinetoplast-to-posterior and anterior lengths, a reduced kinetoplast-to-nucleus distance, and weakened polyglutamylation in the subpellicular microtubule array. Despite these structural changes, α-tubulin detyrosination and EB1 distribution remained unaffected.

### Loss of TTLL6B prolongs cytokinesis and slows cell cycle progression

In order to investigate whether the elongated shape of TTLL6B-deficient cells impacts cytokinesis and cell cycle progression, cell growth was monitored. This revealed an increase in doubling time from 9.6 h in wild-type cells to approximately 11 h in the mutant cell line (Fig. S10C). To further explore this slower growth, a flow cytometry analysis was conducted (Fig. S10D through F). This analysis revealed a slight decrease in cells in the G1 phase of the cell cycle and a relative increase in cells in the G2/M phase, potentially indicating a prolonged cytokinesis. Notably, we did not detect an increase in cells with missegregated nuclei or kinetoplasts, thereby indicating that mitotic fidelity is maintained despite the morphological and growth deficiencies.

### Dosage-dependent phenotype rescue by ectopic TTLL6B expression

To further analyze the role of TTLL6B and the phenotypic consequences of its knockout, we attempted to rescue the wild-type phenotype by expressing TTLL6B through read-through transcription at the tubulin locus as described for TTLL4C. All rescue clones exhibited significantly lower TTLL6B transcript levels compared to wild-type cells. Consequently, a clone exhibiting approximately 20% of the wild-type TTLL6B expression was selected for subsequent analyses (Fig. S11A). Western blotting and immunofluorescence revealed a restoration of PolyE levels in the rescue cell line (Fig. S10A and S11B and C). Additionally, the phenotypic defects observed in the knockout cells were fully reversed in the rescue line (Fig. S11B through F). While the adverse growth effects associated with TTLL6B deficiency were partially mitigated by ectopic expression of the enzyme, the growth rate did not fully recover to wild-type levels (Fig. S10C). However, the proportion of cells in the G2/M phase and the flow cytometry profile in the rescue cell line were restored to wild-type levels (Fig. S10D).

**Fig 6:**
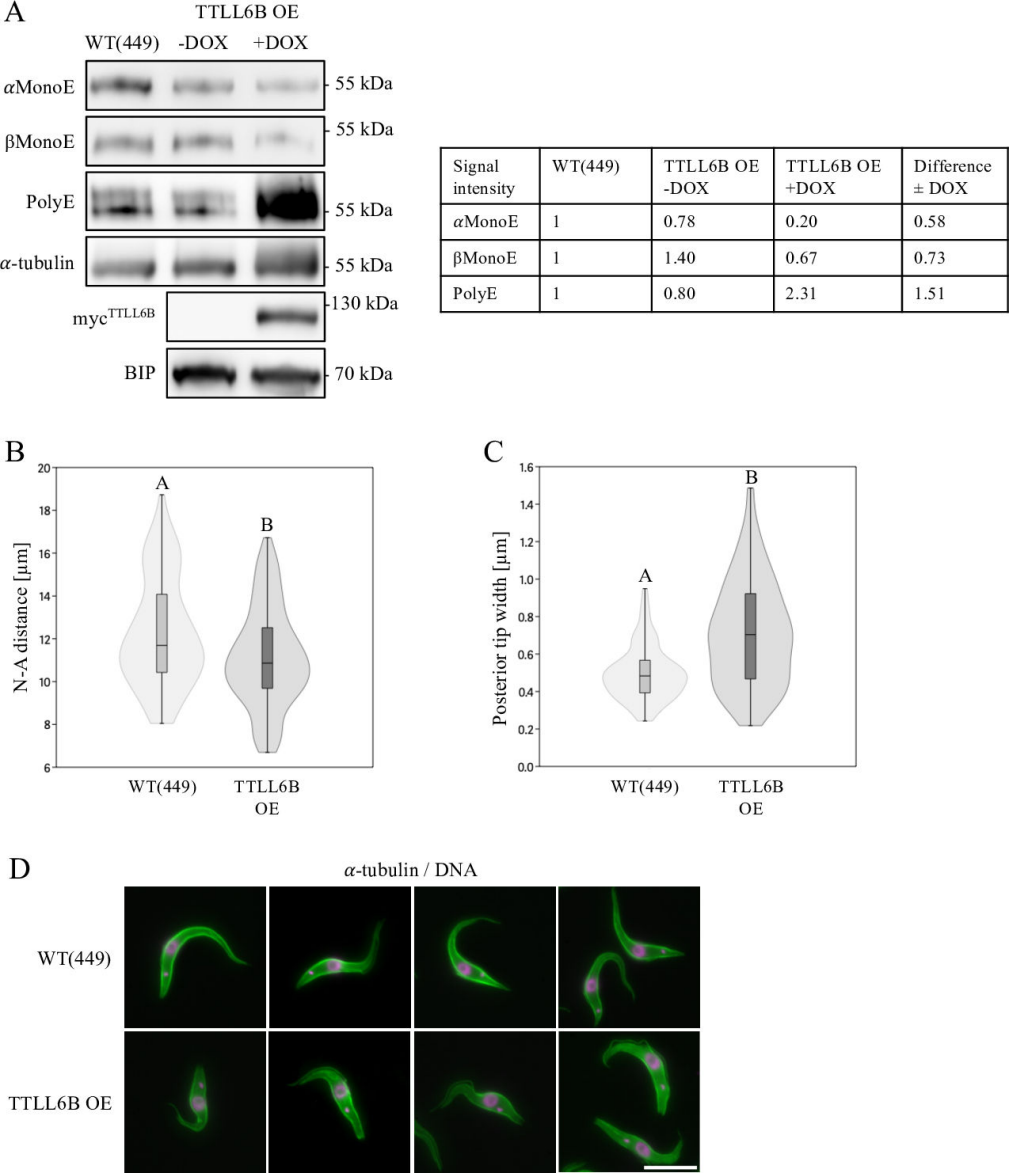
Overexpression of TTLL6B. (**A**) Western blot analysis of cytoskeletal fractions from wild-type (WT, strain 449) and TTLL6B overexpressing (OE) cells. The proteins α-tubulin (TAT1) and BIP were utilized as loading controls. The results presented are representative of three independent experiments. The adjacent table presents the means of three quantified independent western blot results normalized to WT α-tubulin levels. The difference in signal upon doxycycline induction is indicated. (**B**) Quantitative analysis of the nucleus-to-anterior tip (N-A) length distribution in G1-phase WT and TTLL6B OE cells. The N-A length was measured from the center of the nucleus to the anterior tip. (**C**) Assessment of the posterior tip width distribution in G1-phase WT and TTLL6B OE cells. Violin plots illustrate the arithmetic mean, interquartile range, standard error, and density distribution of the data. The sample size (*N*) was 100 cells. Statistical significance between groups is denoted by different letters, with identical letters indicating no significant difference (Mann-Whitney pairwise test, *P* < 0.05). The data presented are representative of three independent experiments. (**D**) Immunofluorescence microscopy of WT and TTLL6B OE cells. Microtubules were visualized using an α-tubulin antibody (TAT1, green), and DNA was stained with DAPI (magenta). Scale bar: 10 µm.

In a subsequent experiment, an inducible version of TTLL6B, driven by a strong promoter, was introduced into a rDNA spacer. The resulting TTLL6B-overexpressing cell line showed a 12-fold increase in TTLL6B expression (Fig. S12A). Western blot analysis confirmed the elevated TTLL6B levels, which led to extensive polyglutamylation of both α- and β-tubulins (Fig. 6A). Although we have identified TTLL6B as an elongase acting mainly on β-tubulin, we speculate that its massive overexpression (see Fig. S12A) leads to a more promiscuous elongation activity. Concurrently, there was a reduction in the signals for monoglutamylated α- and β-tubulin (Fig. 6A). Phenotypically, the TTLL6B-overexpressing cells were significantly shorter and exhibited a blunt posterior end, resembling the phenotype of the TTLL4C knockout (Fig. 6B through D). Notably, there was no detrimental effect on growth; indeed, the overexpressing cells grew slightly better than the parental strain (Fig. S12B). These data suggest that a balance between α- and β-tubulin polyglutamylation is crucial for maintaining cytoskeletal integrity and ensuring proper cytokinesis.

## DISCUSSION

Using western blot analysis, the deletion of TTLL4C resulted in an almost complete loss of αMonoE signal (~95% reduction) and a ~50% decrease in PolyE signal, thus confirming TTLL4C as an α-tubulin-specific initiator. Immunofluorescence analysis revealed a similar pattern, with αMonoE staining absent and PolyE staining clearly detectable. The αMonoE monoclonal antibody, which was generated for this study, specifically recognizes a single glutamate that is linked via an isopeptide bond to residue E445. This residue is the sole polyglutamylation site on α-tubulin in *T. brucei* and mammals (34). A weak residual αMonoE signal observed in *ttll4c^−/−^* cells prompted a mass spectrometric analysis, which demonstrated a marked increase in non-glutamylated α-tubulin. This analysis corroborated the western blot data and confirmed TTLL4C as an initiator. The presence of residual monoglutamylation may be indicative of partial compensation by other TTLL enzymes, consistent with reports of low-level initiase activity in human TTLL6 (17).

Phenotypically, *ttll4c^−/−^* cells exhibited blunt posterior ends and slight shortening, resembling the phenotype of *ttll1^−/−^* mutants (28). EB1 and XMAP215 localization remained unaltered, and growth and motility were only marginally affected. This indicates that TTLL4C primarily contributes to posterior cytoskeletal organization rather than global microtubule dynamics. TTLL1 is likely to function as an α-tubulin elongase, dependent on TTLL4C-mediated initiation to generate the appropriate substrate.

In contrast, TTLL6B depletion resulted in a distinct elongated morphology characterized by a single posterior protrusion and organelle mispositioning, contrasting with the multiple lobular extensions (“glove phenotype”) observed following TTLL6A and TTLL12B depletion (27). Despite overall cell elongation, the nucleus-kinetoplast distance decreased, potentially attributable to mechanical constraints or active repositioning. The phenotype was fully rescued by ectopic expression, confirming its specificity. Biochemically, TTLL6B deletion caused a significant decrease in PolyE signal, particularly on β-tubulin. Therefore, TTLL6B most likely functions as a polyglutamyl elongase with preferential activity toward β-tubulin.

Collectively, these findings support a model in which TTLL4C and TTLL1 act on α-tubulin to maintain a tapered posterior tip, whereas TTLL6A and TTLL6B regulate β-tubulin to prevent excessive microtubule extension. TTLL12B likely functions as an elongase, although its specificity remains unresolved (27). Most phenotypes were restricted to the posterior end, thereby emphasizing its sensitivity to perturbations in polyglutamylation. Future studies employing multiplex gene editing will be required to dissect cooperative TTLL functions and their contribution to cytoskeletal architecture.

The findings on TTLL4C and TTLL6B in *T. brucei* resonate with observations in other systems where polyglutamylation homeostasis is critical for cytoskeletal integrity. In mammals, TTLL1 and TTLL4 have been shown to cooperate with the deglutamylase CCP1 to regulate neuronal microtubules, and their imbalance contributes to neurodegeneration in models of Purkinje cell degeneration (36, 37). Similarly, TTLL6 family enzymes contribute to maintaining axonemal architecture in vertebrates, and their dysfunction is the underlying cause of ciliopathies, such as Joubert syndrome (38, 39). These parallels suggest that the spatial sensitivity of microtubule PTMs, particularly at structural bottlenecks, such as the trypanosome posterior end or neuronal synapses, represents a conserved principle of cytoskeletal regulation (40). Furthermore, polyglutamylation defects are increasingly recognized as drivers of human disease, including retinal dystrophies, such as cone-rod dystrophy caused by CCP5 mutations (41), neurodegenerative disorders (36, 39), and, in mice, male infertility caused by ciliary defects (42, 43).

It is likely that an imbalance in polyglutamylation does not directly result in the observed deficiencies in microtubule organization. A number of studies have shown a direct impact of polyglutamylation on MAP binding and activity of microtubule-associated proteins. The binding of Tau protein to microtubules is enhanced by polyglutamylation on both α- and β-tubulin tails, while Tau association is weakened in a dual knockout of TTLL1 and TTLL7 in mice (25). Kinesin-1 motility is specifically impaired by polyglutamylation on β-tubulin tails, reducing processivity and landing rates (44). Importantly, proteins involved in the depolymerization and polymerization of microtubules are regulated by the polyglutamylation status. The activity of microtubule-severing enzymes katanin and spastin is modulated by microtubule glutamylation, as is the depolymerizing kinesin Kif2C and the microtubule polymerase XMAP215 (45–49). These functional interactions give rise to complex regulatory networks for microtubule turnover and structural integrity, emphasizing that TTLL-mediated PTMs shape microtubule architecture by orchestrating MAP-dependent dynamics. While our observations relating to various phenotypes caused by defective polyglutamylation in *T. brucei* are consistent with data obtained using alternative model organisms, our current understanding of how PTMs influence the function of microtubule-associated proteins remains limited. This will be an important aspect of future research, made challenging, for example, by the fact that *T. brucei* encodes for more than 50 kinesins and many other microtubule-associated proteins, many of which are unique to trypanosomes and related parasites (5, 50, 51).

## MATERIALS AND METHODS

### Trypanosomal cell culture

Procyclic 427 *T. brucei* cells were cultured in SDM-79 medium (Life Technologies, UK) supplemented with 10% fetal bovine serum (Capricorn Scientific, Germany) and 7.5 mg/L hemin (Merck, Germany) at 27°C. Knockout cell lines were selected with puromycin (1 µg/mL) and blasticidin (10 µg/mL); rescue lines with hygromycin (50 µg/mL) and XMAP215-tagged cells with phleomycin (2.5 µg/mL). Overexpression constructs were induced in PC449^TetRBLE^ cells (52), selected with phleomycin (2.5 µg/mL) and hygromycin (50 µg/mL). Cell proliferation was monitored with a CASY cell counter (Roche Innovatis AG, Germany). Statistical data of the cell counts are in Table S3. Population doubling times were calculated with V. Roth’s Doubling Time Computing, available from http://www.doubling-time.com/compute.php. TriTrypDB gene IDs were TTLL4C (Tb927.1.1550) and TTLL6B (Tb927.11.6810).

### Generation of gene knockouts in procyclic *T. brucei*

Knockout constructs were based on modified pBluescript II KS(+) vectors containing additional restriction sites for SbfI, PacI, FseI, and AscI. Puromycin and blasticidin resistance cassettes were amplified from previously described vectors (28) and inserted into the backbone, yielding pKO-Puro^R^ and pKO-BSD, respectively. Approximately 150 bp of the 5′ and 3′ UTRs of the target genes were cloned upstream and downstream of the resistance cassettes to enable homologous recombination. The recombination cassettes were excised with SbfI and AscI and transfected by electroporation using an Amaxa Nucleofector II (Lonza, Germany). Knockout clones were screened by PCR, and correct integration was confirmed by size-shift analysis of the target locus. The resulting strains, Δ*ttll4c::PURO^R^/*Δ*ttll4c::BSD* and Δ*ttll6b::PURO/*Δ*ttll6b::BLA*, are referred to as *ttll4c^−/−^* and *ttll6b^−/−^*. The oligonucleotides utilized in this study are listed in Table S1.

### Ectopic expression of TTLL4C and TTLL6B

Open reading frames of TTLL4C and TTLL6B were amplified from genomic DNA and cloned into the pTag8-tub vector for promoterless integration at the tubulin locus (35), ensuring expression levels comparable to wild type. For overexpression, ORFs were inserted into the doxycycline-inducible pHD1900 (derived from pHD1700^2×myc^) (53), allowing N- or C-terminal tagging under the control of the procyclin promoter.

### Endogenous tagging of *T. brucei* XMAP215

The N-terminus of *Trypanosoma brucei* XMAP215 (Tb927.6.3090) was tagged with mNeonGreen, following established methodology (54). The pPOTv7-phleomycin-3xTy-mNeonGreen-3xTy plasmid served as the template for the tagging construct. Primer sequences required for the tagging procedure were sourced from the TrypTag database (55).

### Real-time quantitative PCR

The mRNA levels of TTLL4C and TTLL6B across different cell lines were quantified using RT-qPCR. Total RNA was extracted using the NucleoSpin RNA Plus Kit (Macherey-Nagel, Germany) and subsequently reverse-transcribed into cDNA with the RevertAid First Strand cDNA Synthesis Kit (Thermo Fisher Scientific). qPCR assays were conducted on a StepOne real-time PCR system (Thermo Fisher Scientific), utilizing Maxima SYBR Green/ROX qPCR master mix (Thermo Fisher Scientific) and 10 ng of cDNA as the template. Primer sequences and the qPCR protocol are provided in Table S2. The constitutively expressed PFR-A gene was used as an endogenous control, and gene expression was calculated using the ΔΔCT method.

### Microscopy

Trypanosomes from exponentially growing cultures were harvested, resuspended in PBS, and allowed to adhere to poly-L-lysine-coated glass slides (VWR, Belgium). Cytoskeletons were extracted with 1% NP-40 in PM buffer (100 mM PIPES-NaOH, 1 mM MgSO₄, pH 6.9). Whole cells or cytoskeletons were fixed in methanol or prefixed in 10% (vol/vol) formaldehyde. Antibody incubations were performed for 1 h each, followed by three PBS washes. DNA was stained with DAPI (1 µg/mL), and slides were mounted in Vectashield (Vector Laboratories, USA). Imaging was performed on a Zeiss Axio Imager.M2 epifluorescence microscope (Carl Zeiss Microscopy GmbH, Germany) equipped with a pco.panda 4.2 M camera (PCO AG, Germany) and VisiView 6.0 imaging software (Visitron Systems GmbH, Germany). Expansion microscopy was performed as described previously (56). To generate cytoskeletal samples, attached cells were extracted with 1% NP-40 in PBS, followed by three PBS washes, prior to fixation. Morphometric analysis used ImageJ2 (v2.14.0/1.54f), and only G1-phase cells (one nucleus, one kinetoplast) were included in measurements. Statistical analysis and data visualization were conducted using the Past4 (v4.15) software package (57). Statistical analysis employed Kruskal–Wallis and Mann–Whitney *U* tests. Figures were created in Microsoft PowerPoint. Images represent at least three independent experiments.

### Isolation and staining of flagella for microscopy

Trypanosomes from exponentially growing cultures were harvested, washed, and resuspended in PBS before being allowed to adhere to poly-L-lysine-coated glass slides (VWR, Belgium). Cytoskeletons were extracted using 0.5% Triton X-100 in PMN buffer (10 mM Na_3_PO_4_, 1 mM MgCl_2_, 150 mM NaCl, pH 7.2). Subpellicular microtubules were subsequently depolymerized by incubating the samples in PMN buffer supplemented with 1 M NaCl, applied in three consecutive extraction steps on ice for 5 min each. The remaining flagella were fixed in cold methanol. Primary and secondary antibody incubations were performed for 1 h each at room temperature, followed by three washes with PBS. Antibodies were used at the following dilutions: 1:7,000 for PolyE and 1:200 for αMonoE.

### Western blotting

Whole-cell lysates were prepared from 4 × 10⁷ cells in 50 µL SDS-PAGE sample buffer (125 mM Tris-HCl, pH 6.8; 5% [vol/vol] glycerol; 4% [vol/vol] SDS; 5% β-mercaptoethanol; bromophenol blue) and heated at 95°C for 10–15 min. For fractionation, cells were lysed in PM buffer supplemented with 1% (vol/vol) NP-40, centrifuged at 20,000 × *g* for 5 min to separate fractions. The cytoskeletal pellet was resuspended in SDS-PAGE buffer and boiled. Flagellar proteins were extracted with 1 M NaCl (6). SDS-PAGE loading amounts varied by antibody: 4 × 10⁶ cells/lane (YL1/2, αCAP5.5); 2 × 10⁵ cells/lane (TAT1, αMonoE), and 5 × 10⁴ cells/lane (PolyE, βMonoE). Proteins were transferred to nitrocellulose (Carl Roth, Germany), probed with primary and HRP-conjugated secondary antibodies, and detected using the ImageQuant LAS-4000 system (GE Healthcare) with Lumigen substrate (Takara Bio, Japan).

### Antibodies

The following antibodies were used: mouse monoclonal IgG anti-myc epitope (clone 9E10*,* DSHB; 1:500 for WB); mouse monoclonal IgG anti-Ty epitope (BB2, gift from Keith Gull, 1:1,000 for IF) (58); mouse monoclonal IgM anti-EB1 (27); rat monoclonal IgG anti-tyrosinated α-tubulin (YL1/2, cat. no. MAB1864, Sigma-Aldrich, Germany; 1:250 for WB, 1:10 for IF); mouse monoclonal IgG anti-BIP (57); mouse monoclonal IgG anti-CAP5.5 (28); rabbit polyclonal anti-polyglutamylated α-/β-tubulin (PolyE; cat. no. AG-25B-0030, Biomol; 1:10,000 for WB, 1:7,000 [TTLL4C] and 1:50,000 [TTLL6B] for IF); rabbit polyclonal anti-monoglutamylated β-tubulin (βMonoE; cat. no. AG-25B-0039, Biomol; 1:5,000 for WB); mouse monoclonal IgG anti-α-tubulin (TAT1) (14); mouse monoclonal anti-monoglutamylated α-tubulin (αMonoE; see below).

### αMonoE antibody production

A monoglutamylated peptide from the C-terminus of trypanosomal α-tubulin [CDMDGE(*-E)EDV] was conjugated to mcKLH (Thermo Fisher Scientific) and used for immunization (50 µg primary, 25 µg boosters) of BALB/c mice (Janvier Labs, France) with Freund’s adjuvant (Thermo Fisher Scientific). Spleen cells were fused to P3X63-Ag8.653, and hybridomas were cultured in HAT-selective OptiMEM (Thermo Fisher Scientific). Supernatants were screened by immunofluorescence and western blot for differential reactivity against WT and *ttll4c*^−/−^ cytoskeletons. Positive clones were subcloned by limiting dilution, and the antibody was typed as IgG1. All procedures complied with local regulations and were approved by the Government of Lower Franconia.

### Cell cycle analysis

For flow cytometry analysis, 6 × 10⁶ cells from exponentially growing *T. brucei* cultures were fixed overnight at 4°C in 3.7% (vol/vol) formaldehyde prepared in SDM-79 medium. The following day, cells were harvested, washed with PBS, and fixed in 70% (vol/vol) ethanol under rotation for 1 h. Subsequently, cells were treated with RNase A (20 µg/mL) and stained with propidium iodide (1 µg/mL) at 37°C for 30 min and analyzed using a Cytomics FC500 flow cytometer (Beckman Coulter).

### Gel electrophoresis

Whole trypanosome cells were washed once in PBS and then dissolved in hot SDS sample buffer. To concentrate samples, protein precipitation was performed overnight at −20°C with a fourfold volume of acetone. Pellets were washed with acetone at −20°C. Precipitated proteins were dissolved in NuPAGE LDS sample buffer (Life Technologies), reduced with 50 mM DTT at 70°C for 10 min, and alkylated with 120 mM iodoacetamide at room temperature for 20 min. Separation was performed on NuPAGE Novex 4%–12% Bis-Tris gels (Life Technologies) with MOPS running buffer according to the manufacturer’s instructions. Gels were washed three times for 5 min with water, stained for 1 h with Simply Blue Safe Stain (Life Technologies), and washed with water for 1 h.

### In-gel digestion

The gel sections containing the two bands for α- and β-tubulin were excised from the gel and destained with 30% acetonitrile in 0.1 M NH_4_HCO_3_ (pH 8), shrunk with 100% acetonitrile, and dried in a vacuum concentrator (Concentrator 5301, Eppendorf, Germany). Digests were performed with 0.25 µg ProAlanase (Promega, Mass Spec Grade) per gel band overnight at 37°C in 0.2% formic acid. The digest was terminated by heating the sample at 90°C for 10 min. After removing the supernatant, peptides were extracted from the gel slices with 5% formic acid, and the extracted peptides were pooled with the supernatant.

### NanoLC-MS/MS analysis

NanoLC-MS/MS analyses were performed on an Orbitrap Fusion (Thermo Scientific) equipped with a PicoView Ion Source (New Objective) and coupled to an EASY-nLC 1000 (Thermo Scientific). Peptides were loaded on a trapping column (2 cm × 150 µm ID, PepSep) and separated on a capillary column (30 cm × 150 µm ID, PepSep), both packed with 1.9 µm C18 ReproSil and separated with a 30-min linear gradient from 3% to 30% acetonitrile and 0.1% formic acid and a flow rate of 500 nL/min. Both MS and MS/MS scans were acquired in the Orbitrap analyzer with a resolution of 60,000 for MS scans and 30,000 for MS/MS scans. A mixed ETD/HCD method was used. HCD fragmentation was applied with 35% normalized collision energy. For ETD, calibrated charge-dependent ETD parameters were applied. A top speed data-dependent MS/MS method with a fixed cycle time of 3 s was used. Dynamic exclusion was applied with a repeat count of 1 and an exclusion duration of 10 s. Peptides with charge states 2–3 were selected for HCD fragmentation, and charge states 3–6 were selected for ETD fragmentation. Singly charged precursors were excluded from selection. Minimum signal threshold for precursor selection was set to 50,000 for HCD and 100,000 for ETD. Predictive AGC was used with a target value of 4 × 10^5^ for MS scans and 5 × 10^4^ for MS/MS scans. EASY-IC was used for internal calibration.

### Mass spectrometry data analysis

Database search was performed against the UniProt Reference proteome of *Trypanosoma brucei* strain 927 (UP000008524, 8587 protein entries) with PEAKS Xpro software (Bioinformatics Solutions Inc.) with the following parameters: peptide mass tolerance: 10 ppm, MS/MS mass tolerance: 0.02 Da, enzyme: “ProAlanase,” variable modifications: acetylation (protein N-term), oxidation (M); carbamidomethylation (C), Pyro-glu from Q, as well as glutamylation modifications with one to four glutamate residues (E).

### Peptide synthesis and purification

The linear and branched peptides (CDMDGEEDV and CDMDGE^E^EDV, single letter code, ^E^ denotes branched glutamic acid via isopeptide bond) were synthesized on a 2-chloro-trityl-chloride-polystyrene resin: the couplings of the amino acids were either carried out manually or using a peptide synthesizer. In the case of the branched peptides, an on-resin deallylation of the glutamate was performed on the tetrapeptide stage, and the branching glutamate was inserted afterward. After deprotection, the peptides 1–4 were purified by RP-HPLC. The cysteine-bearing peptides 1 and 3 were conjugated with maleimide-activated mcKLH (Imject, Thermo Scientific, Germany) according to the manufacturer’s instructions. Details of synthesis are provided in the Supplemental material.

## Data Availability

All relevant data and details of resources can be found within the article and its Supplemental material.

## SUPPLEMENTAL MATERIAL

Supplemental material msphere.00909-25-s0001.pdf Supplemental tables, text, and figures.

ASM does not own the copyrights to Supplemental Material that may be linked to, or accessed through, an article. The authors have granted ASM a non-exclusive, world-wide license to publish the Supplemental Material files. Please contact the corresponding author directly for reuse.
