# Supplementary material for: Tubulin polyglutamylases TTLL4C and TTLL6B are essential for maintaining cytoskeletal integrity in Trypanosoma brucei
Source: mSphere. 2026 Apr 30;11(5):e00909-25. doi: 10.1128/msphere.00909-25 (PMC13203978; doi:10.1128/msphere.00909-25)
Supplement: Supplemental material — Supplemental tables, text, and figures. [file msphere.00909-25-s0001.pdf]

Supplementary information

Tubulin polyglutamylases TTLL4C and TTLL6B are essential for maintaining cytoskeletal integrity in Trypanosoma brucei

Lucas Brehm[1], Moritz Röder[2], Stephanie Lamer[3,4], Marinus Thein[1], Andreas Schlosser[3,5], Carlo Unverzagt[2], Klaus Ersfeld[1,*]

Suppl. Table 1: Oligonucleotides

modification of pBluescript II	pKO 5' mod for	CGCGCTACCTGCAGGGCGCTATTAATTAAGCGCTAT
	pKO 5' mod rev	CTAGATAGCGCTTAATTAATAGCGCCCTGCAGGTAGCGCGAGCT
	pKO 3' mod for	TCGAGTATTAGGGCCGGCCTATTAGGGCGCGCCTATTAGGGTAC
	pKO 3' mod rev	CCTAATAGGCGCGCCCTAATAGGCCGGCCCTAATAC
TTLL4C Homology arm amplification Tb927.1.1550	5' homology for	GACCTGCAGGCGGCGTTACATTACATGGAGAAACCG
	5' homology rev	GCGTTAATTAAGCATCACTTTGAAGCCTTGGCAGTGGTGAG
	3' homology for	CGATGGCCGGCCCCGAGTGAAATTTACGGACATGC
	3' homology rev	GGGCGCGCCTTGAGCCCAACACCACAACGGCTACATCC
TTLL4C PCR analysis	TTLL4C 5' KO Seq	CACCTCCACGAAGCGTCGTATGAG
	3' homology rev	GGGCGCGCCTTGAGCCCAACACCACAACGGCTACATCC
TTLL4C qPCR analysis	qTTLL4C for	CACGCTCAGCTTTACGATGC
	qTTLL4C rev	ATGAGAAGGCTCCCCAGCTA
TTLL4C Rescue	TTLL4C Ex for	TATGGCCGGCCATGACAGACGGCGTTACATTAC
	TTLL4C Ex rev	TATGGCGCGCCTTAAGAAGCCCACGATGG
TTLL4C Overexpression	TyTTLL4C OE for	TATGGCCGGCCG ACAGACGGCGTTACATTAC
	TyTTLL4C OE rev	TATGGCGCGCCTTAAGAAGCCCACGATGG
TTLL6B Homology arm amplification Tb927.11.6810	5' homology for	CGCCTGCAGGGGATGAGGAACATGATGGAG
	5' homology rev	GCTTAATTAAGCGAAGGATTTACTGAGAGGG
	3' homology for	CGAGGCCGGCCGGTGGTGACAGTGACAACAG
	3' homology rev	ATGGCGCGCCCTTCTCTTTCCGCATTCTCTTG
TTLL6B PCR analysis	TTLL6B 5' KO Seq	CCAATGTACAGAGCACATTC
	3' homology rev	ATGGCGCGCCCTTCTCTTTCCGCATTCTCTTG
TTLL6B qPCR analysis	qTTLL6B for	TGCCAGGGCCGAGGAATTAT
	qTTLL6B rev	CGCGCAGATCAAACTTCCGT
TTLL6B Rescue + Overexpression	TTLL6B Ex for	TATGGCCGGCCATGAGGAACATGATGGAGCC
	TTLL6B Ex rev	TATGGCGCGCCCCTCATCCAAATGGAATGAATC
qPCR normalization	qPFR-A for	CGTTGGAGATGTTTGGACCT
	qPFR-A rev	GCACGGTACTCCACCATCTT

Suppl. Table 2: qPCR thermal cycling protocol

Step	Temperature, time	Number of cycles
Initial denaturation	95°C, 30 s	1
Denaturation	95°C, 15 s	
Annealing	55°C, 30 s	40
Extension	68°C, 1 kbp/min	
Final extension	68°C, 5 min	1
Thermal melting	95°C, 15 s	1
	60°C, 60 s	
	+0.3°C/20 s to 95°C	
	95°C, 15 s	

Suppl. Table 3: Standard Deviation Growth curves [cells x 10^6/mL]	Day 1	Day 2	Day 3	Day 4	Day 5	Day 6
WT(427)	0	0.02595	0.58066	3.69282	23.27897	155.05988
ttll4c -/-	0	0.01517	0.54016	3.20715	40.60728	237.22613
ttll6b -/-	0	0.06773	0.53475	1.84304	6.63777	31.77644
TTLL4C Rescue no tag	0	0.05074	0.54569	1.54367	11.07409	35.21635
TTLL6B Rescue myc	0	0.12129	0.07760	0.31776	8.39063	16.91739
WT(449)	0	0.03333	0.14245	2.33583	4.27682	76.68047
WT (449) +DOX	0	0.22258	0.67615	3.33970	21.76209	58.14686
TTLL6B OE	0	0.17412	0.48137	1.51323	14.71960	154.61784
TTLL6B OE +DOX	0	0.21891	1.40350	1.64619	14.28834	49.73450

Supplementary Information: Synthesis of peptides

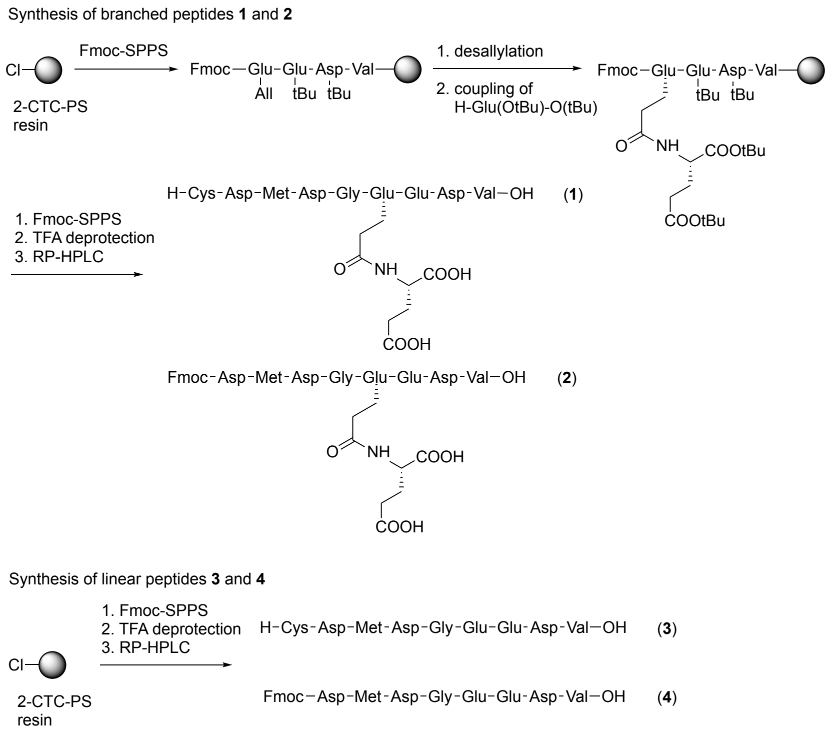

Figure 1 Synthesis of Peptides **1-4**

Supplementary part:

Experimental

General procedures:

1. Preparation of the resin

2-Chloro-Trityl-Polystyrene (2-CTC) resin (1.6 mmol/g, 1eq) was swollen in CH_2Cl_2 (50 mg/mL) for 1 h. A solution of Fmoc-Val-OH (1 eq) and N,N-diisopropylethylamine (DIPEA) (3 eq) in CH_2Cl_2 was added to the preswollen resin and shaken at room temperature overnight. The loaded resin was then washed three times with CH_2Cl_2 (50 mg/mL) and the unreacted sites were quenched using a mixture of CH_2Cl_2/MeOH/DIPEA (17/2/1) (50 mg/mL) under shaking at room temperature for 10 min (3 repetitions). Subsequently the resin was washed three times with CH_2Cl_2 (50 mg/mL). Afterwards the degree of loading was determined. Therefor three samples of resin (around 1mg each) were dried in high vacuum and then treated with 1.5 mL of a 20% Piperidine/DMF solution. The three mixtures were shaken at room temperature for 20 minutes and then centrifugated for 5 min at 20000g. Then 750 µL of the supernatant were diluted with 750µL of the 20% Piperidine/DMF solution and the UV Absorption of each Probe was measured at 290nm. The degree of loading (B_H) could be then determined using the following formula:

$$B_H\left[\frac{mmol}{g}\right] = \frac{A_{290} \cdot V \cdot DF}{m_{resin}[mg] \cdot \varepsilon \cdot d} \cdot 1000$$

A_{290}= measured absorption at 290nm Wavelength, V = volume of the used sample, DF = dilution factor, ε = extinction coefficient (here: 5253 m^3/mol), *d* = thickness of the cuvette

2. Elongation of the peptides

The peptide was elongated using Fmoc solid-phase peptide synthesis either on a Tribute peptide synthesizer (PTI) or manually. The Fmoc-amino acids (5 eq) were coupled in 1.5 mL DMF for 90 min at room temperature in with HCTU (4.8 eq) and DIPEA (6 eq). The Fmoc groups were cleaved using a solution of 20% piperidine in DMF. The Deprotection was monitored by UV-Absorption. In the case of peptides **2** and **4** no Fmoc deprotection was carried out after the coupling of the third aspartate. In the case of peptides **1** and **3** Fmoc deprotection was carried out and the terminal cysteine was coupled as Boc-Cys(Trt)-OH in 1.5 mL DMF for 180 min with HOBt (5eq) and DIC (5eq).

3. On-Resin desallylation of Glu(OAll) and coupling of H-Glu(OtBu)-O(tBu)

The on-resin desallylation was carried out at the tetrapeptide stage.[1,2] The resin was swollen in dry CH_2Cl_2 (50mg/mL) for 1 h. $Pd(PPh_3)_4$ (1.5 eq), DIPEA (2 eq) and Phenylsilane (80 eq) were dissolved in dry CH_2Cl_2, added to the preswollen resin (50mg/mL) and shaken under light exclusion at room temperature. After 1 h the now blackish resin was washed three times with CH_2Cl_2 (50mg/mL) and three times with DMF (50mg/mL) and then swollen for 30 min in DMF (50mg/mL). H-Glu(OtBu)-O(tBu) (5 eq), HCTU (4.8 eq) and DIPEA (6 eq) were dissolved in DMF, added to the preswollen resin (50mg/mL) and the mixture was shaken at room temperature. After 1 h the resin was washed three times with DMF and three times with CH_2Cl_2 (50mg/mL each).

4. Cleavage and purification

The Resin was treated with a mixture of TFA/TIS/H_2O (96/2/2) (25mg/mL) in a fritted syringe for 3 h at room temperature. The filtrate was dried *in vacuo*, precipitated with a tenfold amount of diethyl ether for 20 min at -24 C and the suspension then centrifugated for 5 min at 5000g. This procedure was repeated three times. The crude residue was dried in high vacuum and then dissolved in a mixture of H_2O and MeCN + 0.1% TFA (2 mg/mL) and loaded onto a reversed phase column (Ascentis C18, 250x21.2 mm, 5 µm, 10 mL/min, 6 CV). After purification the fractions containing the desired pure peptides were united and lyophilized.

5. Analytics

<u>RP-UHPLC-ESI-ToF-MS</u>

Analytical separations were carried out on an Acquity UPLC H-Class UHPLC system from Waters with a diode array detector (detection λ = 200 - 300 nm; bandwidth 1.2 nm; flow cell: 500 nL, 10 mm beam path), which was coupled to a Micromass LCT ESI-ToF mass spectrometer. H_2O + 0.1% HCOOH (A) and MeCN + 0.1% HCOOH (B) were used as running media. Elution was performed at 0.5 mL/min initially with the starting concentration of (B) for 1.25 min, followed by a linear gradient to the target concentration of (B) over 5 min. The RP columns were thermostatted to 40°C or 70°C. RP columns used were: YMC-UltraHT Hydrosphere C18 (50 x 2 mm, 2 µm, 120 Å) and the YMC-Triart C18 (50 x 2 mm, 1.9 µm, 120 Å).

<u>RP-UHPLC-ESI-hybrid quadrupole-Orbitrap-MS</u>

High-resolution mass spectra were recorded as direct inlet on a Q Exactive Orbitrap mass spectrometer with an ESI source and coupled RP-UHPLC (UltiMate 3000 UHPLC) from Thermo-Fisher Scientific. H2O + 0.1% HCOOH (A) and MeCN + 0.1% HCOOH (B) were used as running media

6. Conjugation of peptides 1 and 2 with mcKLH

mcKLH was suspended in 200 µL H_2O and the respective peptide was added. The Suspension was mixed carefully and incubated at room temperature for 2 h. The suspension was then transferred into a 500 µL Slide-A-Lyzer dialysis cassette and dialysed against PBS buffer. The buffer was exchanged after 2 h and again after 16 h.

Synthesis of the Peptides:

H-Cys-Asp-Met-Asp-Gly-Glu(Glu)-Glu-Asp-Val-OH (1)

2-CTC-PS resin (220 mg, B_H = 1.6 mmol/g) was placed in a fritted syringe and preswelled with 4 mL of dry CH_2Cl_2 for 1h at RT. Then Fmoc-Val-OH (120 mg, 0,352 mmol) and DIPEA (182 µL, 1 mmol) were dissolved in 4 mL of dry CH_2Cl_2, added to the preswelled resin and shaken at room temperature overnight. The loaded resin was then washed three times with 4 mL of CH_2Cl_2 and the unreacted sites were quenched using a mixture of CH_2Cl_2/MeOH/DIPEA (17/2/1, v/v/v, 4mL) under shaking at room temperature for 10 min (3 repetitions). Subsequently the resin was washed three times with CH_2Cl_2 (4 mL). Afterwards the degree of loading was determined. (B_H = 0.6 mmol/g). The next three amino acids were coupled manually (table 1). After every coupling the resin was washed three times with 4 mL of DMF, Fmoc deprotected using 4 mL of a 20% piperidine/DMF solution (4 repetitions) and washed again three times with 4 mL of DMF. On the tetrapeptide stage no Fmoc deprotection was performed. The resin was dried in high vacuum and split. 120 mg of dried resin were transferred into a new fritted syringe and preswelled in 2 mL of dry CH_2Cl_2. Then $Pd(PPh_3)_4$ (126 mg, 0,108 mmol), DIPEA (25µL, 0,144 mmol), and Phenylsilane (720µl, 5,76 mmol) were dissolved in 2 mL of dry CH_2Cl_2 and added to the preswelled resin. The mixture was shaken at RT for 1 h under light exclusion. The now blackish resin was then washed with CH_2Cl_2 (3x) and DMF (3x). Afterwards H-Glu(OtBu)-O(tBu) (106 mg, 0,36 mmol), HCTU (144 mg, 0,345 mmol) and DIPEA (75µL, 0,432 mmol) were dissolved in 2 mL DMF and added to the resin. The mixture was shaken at RT for 1h, washed with DMF(3x) and a Fmoc deprotection was performed as described before. The remaining amino acids were then coupled again manually (table1)

Table1: Coupling conditions of the Fmoc-SPPS of peptide **1**.

Position	Building block	n [mmol]	m [mg]	Coupling conditions	Coupling time [min]
Asp-8	Fmoc-Asp(OtBu)-OH	0,66	272	[a]	60
Glu-7	Fmoc-Glu(OtBu)-OH	0,66	281	[a]	60
Glu-6	Fmoc-Glu(OAll)-OH	0,66	270	[a]	60
Gly-5	Fmoc-Gly(Dmb)-OH	0,36	161	[b]	120
Asp-4	Fmoc-Asp(OtBu)-OH	0,36	148	[a]	180
Met-3	Fmoc-Met-OH	0,36	134	[a]	60
Asp-2	Fmoc-Asp(OtBu)-OH	0,36	148	[a]	60
Cys-1	Boc-Cys(Trt)-OH	0,26	167	[c]	180

[a] The amino acid and HCTU (262 mg, 0.633 mmol) are dissolved in 4 mL DMF and DIPEA (136 µL, 0,792 mmol) is added. The solution is then added to the preswelled resin.

[b] The amino acid and PyBOP (180 mg, 0.345 mmol) are dissolved in 2 mL DMF and DIPEA (75 µL, 0,432 mmol) is added. The solution is then added to the preswelled resin.

[c] The amino acid and HOBt (53 mg, 0.345 mmol) are dissolved in 2 mL DMF and DIC (54 µL, 0,345 mmol) is added. The solution is then added to the preswelled resin.

After the completion of the Fmoc-SPPS about 20mg of resin were treated with 2 mL of a solution of 96/2/2 (v/v/v) TFA/TIS/H_2O in a fritted syringe at rt for 3h. The filtrate was then precipitated with a tenfold amount of Et_2O, and cooled for 30 min at -24 °C. After centrifugation (5200 rpm, 5 min) the supernatant was removed, and the precipitation was repeated two more times.

The crude residue (20 mg) was dried in high vacuum, dissolved in 5% MeCN/H_2O + 0.1% TFA (20 mL) and purified via HPLC (Ascentis C18 250x21 mm, Gradient: 5-25 MeCN/H_2O + 0.1% TFA in 7 CV, 10 mL/min) giving a yield of 1.2 mg (**1**) (1 µmol, 9%).

LCMS analytics for Peptide **1**:

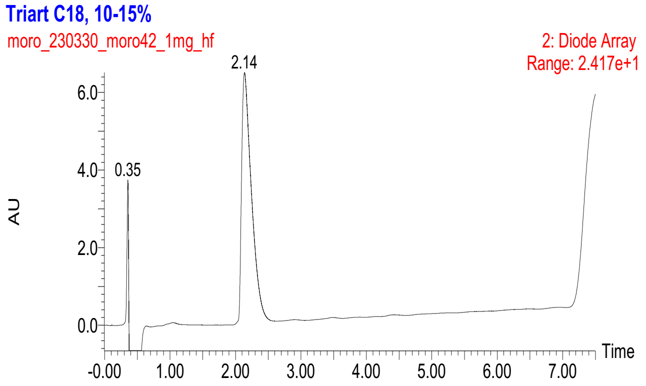

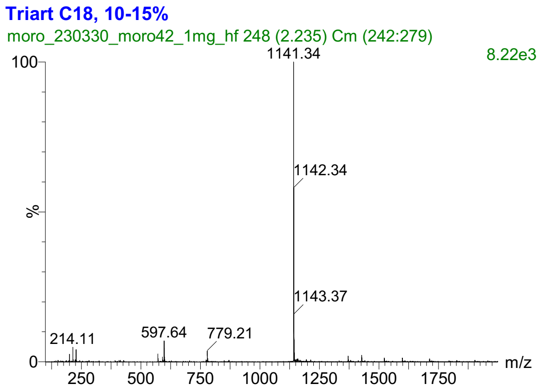

Fmoc-Asp-Met-Asp-Gly-Glu(Glu)-Glu-Asp-Val-OH (2)

220 mg 2-CTC-PS resin were prepared as described for the synthesis of peptide **1** and the degree of loading (B_H = 0.2 mmol/g) was determined. This time the Fmoc-SPPS of the next three amino acids was carried out using a peptide synthesizer (table 2). The resin was then transferred into a new fritted syringe and preswelled in 4 mL of dry CH_2Cl_2. Then $Pd(PPh_3)_4$ (76 mg, 66 µmol), DIPEA (16 µL, 88 µmol), and Phenylsilan (437 µl, 3,52 mmol) were dissolved in 4 mL of dry CH_2Cl_2 and added to the preswelled resin. The mixture was shaken at RT for 1 h under light exclusion. The now blackish resin was then washed with 4 mL CH_2Cl_2 (3x) and 4 mL DMF (3x). Afterwards H-Glu(OtBu)-O(tBu) (65 mg, 0,22 mmol), HCTU (82 mg, 0,198 mmol) and DIPEA (47 µL, 0,264 mmol) were dissolved in 4 mL DMF and added to the resin. The mixture was shaken at RT for 1h and then washed with 4 mL DMF (3x). The remaining amino acids were coupled again in a peptide Synthesizer (table 2)

Table2: Coupling conditions of the Fmoc-SPPS of peptide **2**.

Position	Building block	n [mmol]	m [mg]	Coupling conditions	Coupling time [min]
Asp-8	Fmoc-Asp(OtBu)-OH	0,22	91	[a]	60
Glu-7	Fmoc-Glu(OtBu)-OH	0,22	94	[a]	60
Glu-6	Fmoc-Glu(OAll)-OH	0,22	90	[a]	60
Gly-5	Fmoc-Gly(Dmb)-OH	0,22	98	[b]	120
Asp-4	Fmoc-Asp(OtBu)-OH	0,22	91	[a]	180
Met-3	Fmoc-Met-OH	0,22	82	[a]	60
Asp-2	Fmoc-Asp(OtBu)-OH	0,22	91	[a]	60

[a] The amino acid and HCTU (87 mg, 0.211 mmol) are dissolved in 4 mL DMF and DIPEA (47 µL, 0,264 mmol) is added. The solution is then added to the preswelled resin.

[b] The amino acid and PyBOP (110 mg, 0.211 mmol) are dissolved in 4 mL DMF and DIPEA (47 µL, 0,264 mmol) is added. The solution is then added to the preswelled resin.

After the completion of the Fmoc-SPPS about 60 mg of resin were treated with 6 mL of a solution of 96/2/2 (v/v/v) TFA/TIS/H_2O in a fritted syringe at rt for 3h. The filtrate was then precipitated with a tenfold amount of Et_2O, and cooled for 30 min at -24 °C. After centrifugation (5200 rpm, 5 min) the supernatant was removed, and the precipitation was repeated two more times. The crude residue (40 mg) was dried in high vacuum, dissolved in 30% MeCN/H_2O + 0.1% TFA (40 mL) and purified via HPLC (Ascentis C18 250x21 mm, Gradient: 30-40 MeCN/H_2O + 0.1% TFA in 7 CV, 10 mL/min) giving a yield of 0.2 mg (**2**) (158 nmol, 1,3%).

LCMS analytics for Peptide **2**:

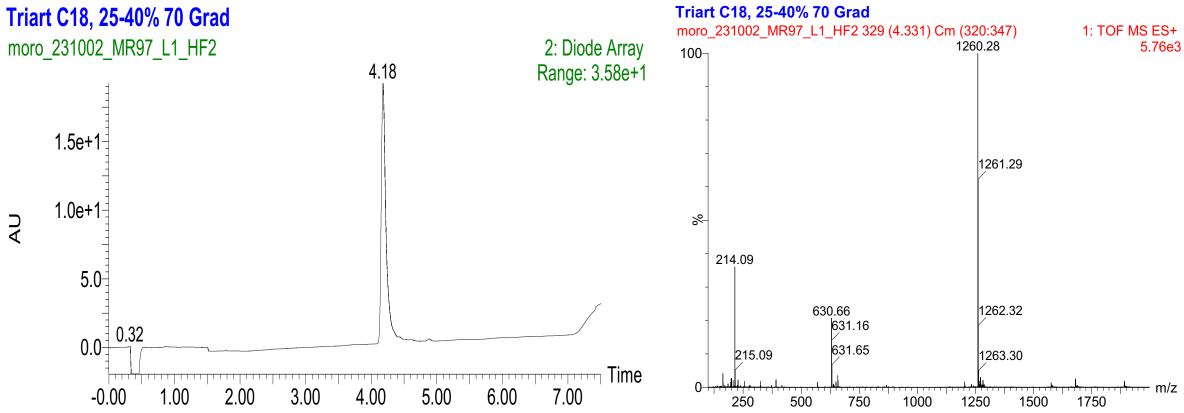

Fmoc-Asp-Met-Asp-Gly-Glu-Glu-Asp-Val-OH (4)

220 mg 2-CTC-PS resin were prepared as described for the synthesis of peptide **1** and the degree of loading (B_H = 0.18 mmol/g) was determined. The rest of the Fmoc-SPPS was carried out using a peptide synthesizer (table 3).

Table3: Coupling conditions of the Fmoc-SPPS of peptide **4**.

Position	Building block	n [mmol]	m [mg]	Coupling conditions	Coupling time [min]
Asp-8	Fmoc-Asp(OtBu)-OH	0,2	81	[a]	60
Glu-7	Fmoc-Glu(OtBu)-OH	0,2	84	[a]	60
Glu-6	Fmoc-Glu(OAll)-OH	0,2	80	[a]	60
Gly-5	Fmoc-Gly(Dmb)-OH	0,2	89	[b]	120
Asp-4	Fmoc-Asp(OtBu)-OH	0,2	81	[a]	180
Met-3	Fmoc-Met-OH	0,2	74	[a]	60
Asp-2	Fmoc-Asp(OtBu)-OH	0,2	981	[a]	60

[a] The amino acid and HCTU (78 mg, 0.191 mmol) are dissolved in 4 mL DMF and DIPEA (42 µL, 0,237 mmol) is added. The solution is then added to the preswelled resin.

[b] The amino acid and PyBOP (99 mg, 0.191 mmol) are dissolved in 4 mL DMF and DIPEA (42 µL, 0,237 mmol) is added. The solution is then added to the preswelled resin.

After the completion of the Fmoc-SPPS about 30 mg of resin were treated with 3 mL of a solution of 96/2/2 (v/v/v) TFA/TIS/H_2O in a fritted syringe at rt for 3h. The filtrate was then precipitated with a tenfold amount of Et_2O, and cooled for 30 min at -24 °C. After centrifugation (5200 rpm, 5 min) the supernatant was removed, and the precipitation was repeated two more times. The crude residue (20 mg) was dried in high vacuum, dissolved in 30% MeCN/H_2O + 0.1% TFA (20 mL) and purified via HPLC (Ascentis C18 250x21 mm, Gradient: 37-42 MeCN/H_2O + 0.1% TFA in 7 CV, 10 mL/min) giving a yield of 4.1 mg (**2**) (3,62 µmol, 67%).

LCMS analytics for Peptide **4**:

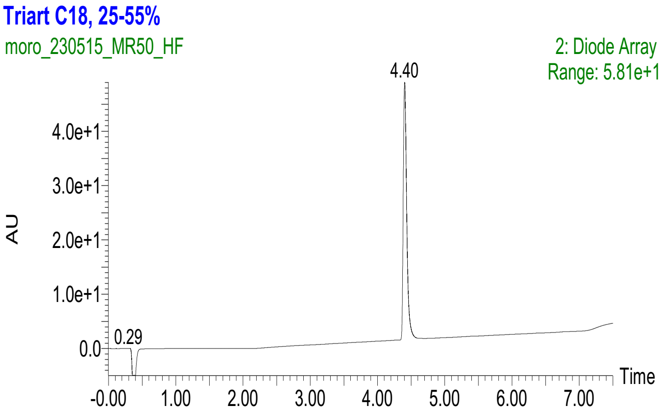

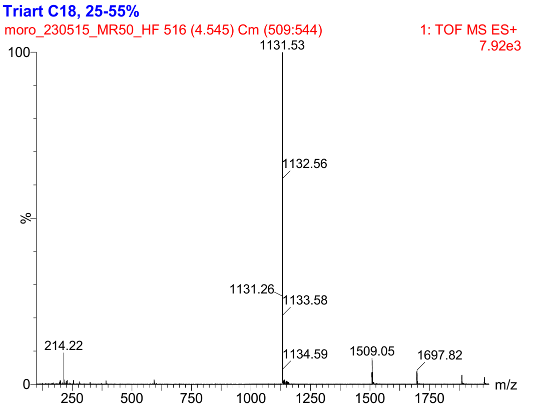

H-Cys-Asp-Met-Asp-Gly-Glu-Glu-Asp-Val-OH (3)

80 mg dried resin from the preparation of peptide (**4**) were preswelled in 2 mL DMF, Fmoc deprotected using 2 mL of a 20% piperidine/DMF solution (4 repetitions) and washed three times with 2 mL of DMF. Boc-Cys(Trt)-OH (33mg, 70 µmol), HOBt (11 mg, 69µmol) and DIC (11µL, 69 µmol) were dissolved in 2 mL DMF and added to the resin. The mixture was shaken at rt for 3 h and the resin then washed three times with DMF (2mL) and three times with CH_2Cl_2. About 30 mg of resin were treated with 3 mL of a solution of 96/2/2 (v/v/v) TFA/TIS/H_2O in a fritted syringe at rt for 3h. The filtrate was then precipitated with a tenfold amount of Et_2O, and cooled for 30 min at -24 °C. After centrifugation (5200 rpm, 5 min) the supernatant was removed, and the precipitation was repeated two more times. The crude residue (20 mg) was dried in high vacuum, dissolved in 5% MeCN/H_2O + 0.1% TFA (20 mL) and purified via HPLC (Ascentis C18 250x21 mm, Gradient: 5-25 MeCN/H_2O + 0.1% TFA in 7 CV, 10 mL/min) giving a yield of 2.9 mg (**2**) (2,86 µmol, 57%).

LCMS analytics for Peptide **3**:

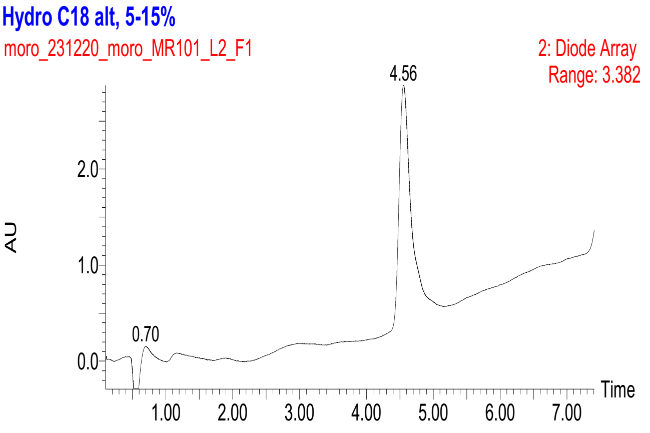

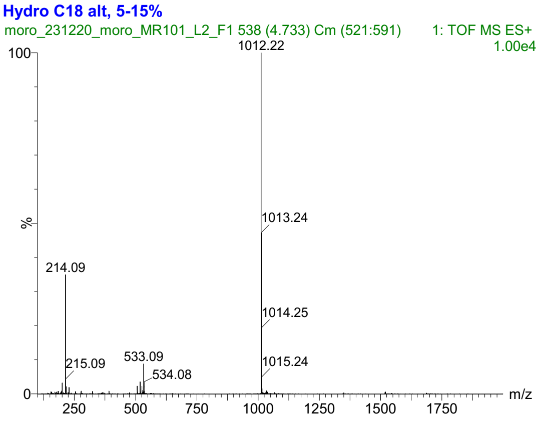

References:

[1] M. Dessolin, M.-G. Guillerez, N. Thieriet, F. Guibe, A. Loffet, *Tetrahedron Lett.* **1995**, *36*, 5741.
[2] Ullmann, V., Rädisch, M., Boos, I., Freund, J., Pöhner, C., Schwarzinger, S., Unverzagt, C. *Angew. Chem., Int. Ed.*, **2012**,*51(46)*, 11566.

HRMS analytics

Peptide **1**: [M+H]$^+$ calculated: 1141.3660 (bottom), [M+H]$^+$ found: 1142.3631 (top)

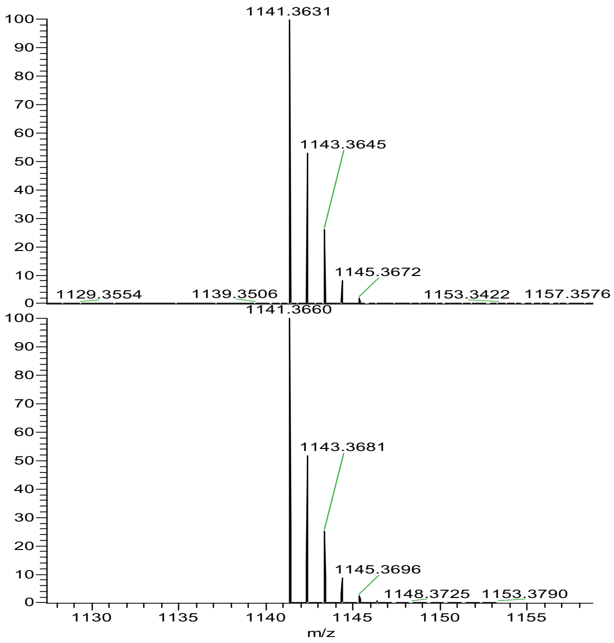

Peptide **2**: [M+H]$^+$ calculated: 1260.4249 (bottom), [M+H]$^+$ found: 1260.4206 (top)

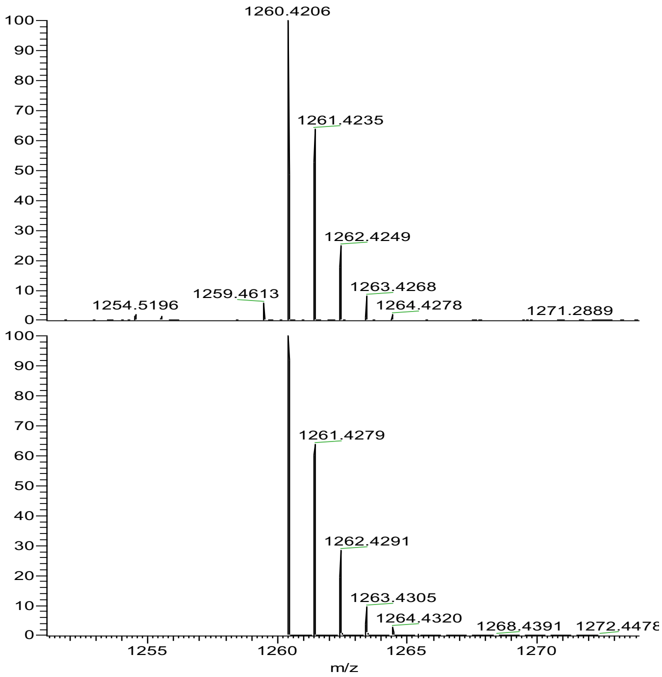

Peptide **3**: $[M+H]^+$ calculated: 1131.3823 (bottom), $[M+H]^+$ found: 1131.3766 (top)

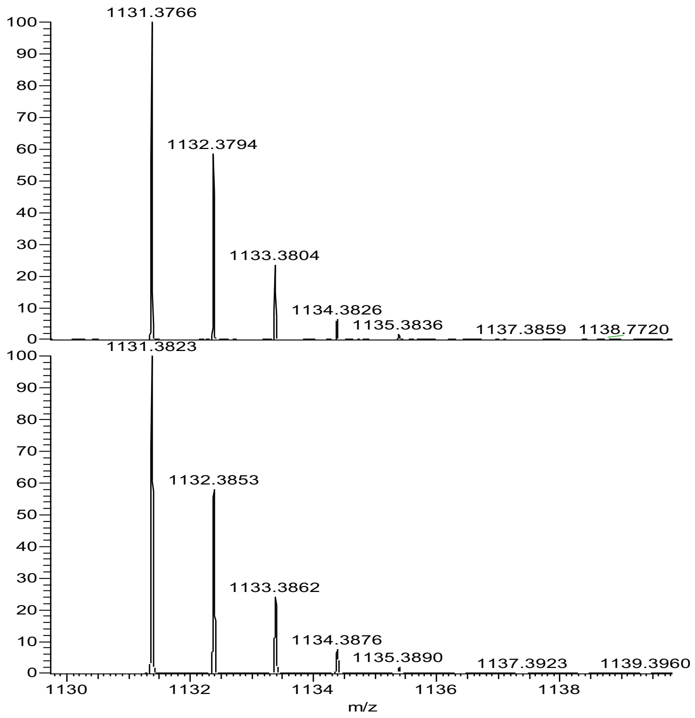

Peptide **4**: $[M+H]^+$ calculated: 1012.3234 (bottom), $[M+H]^+$ found: 1012.3178 (top)

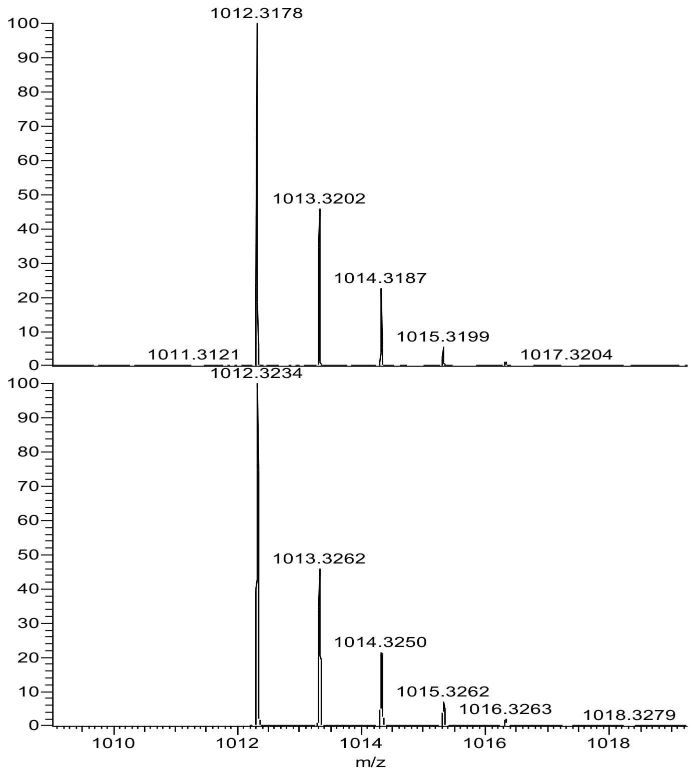
1012.3178
1013.3202
1014.3187
1015.3199
1011.3121
1017.3204
1012.3234
1013.3262
1014.3250
1015.3262
1016.3263
1018.3279
1010
1012
1014
1016
1018
m/z

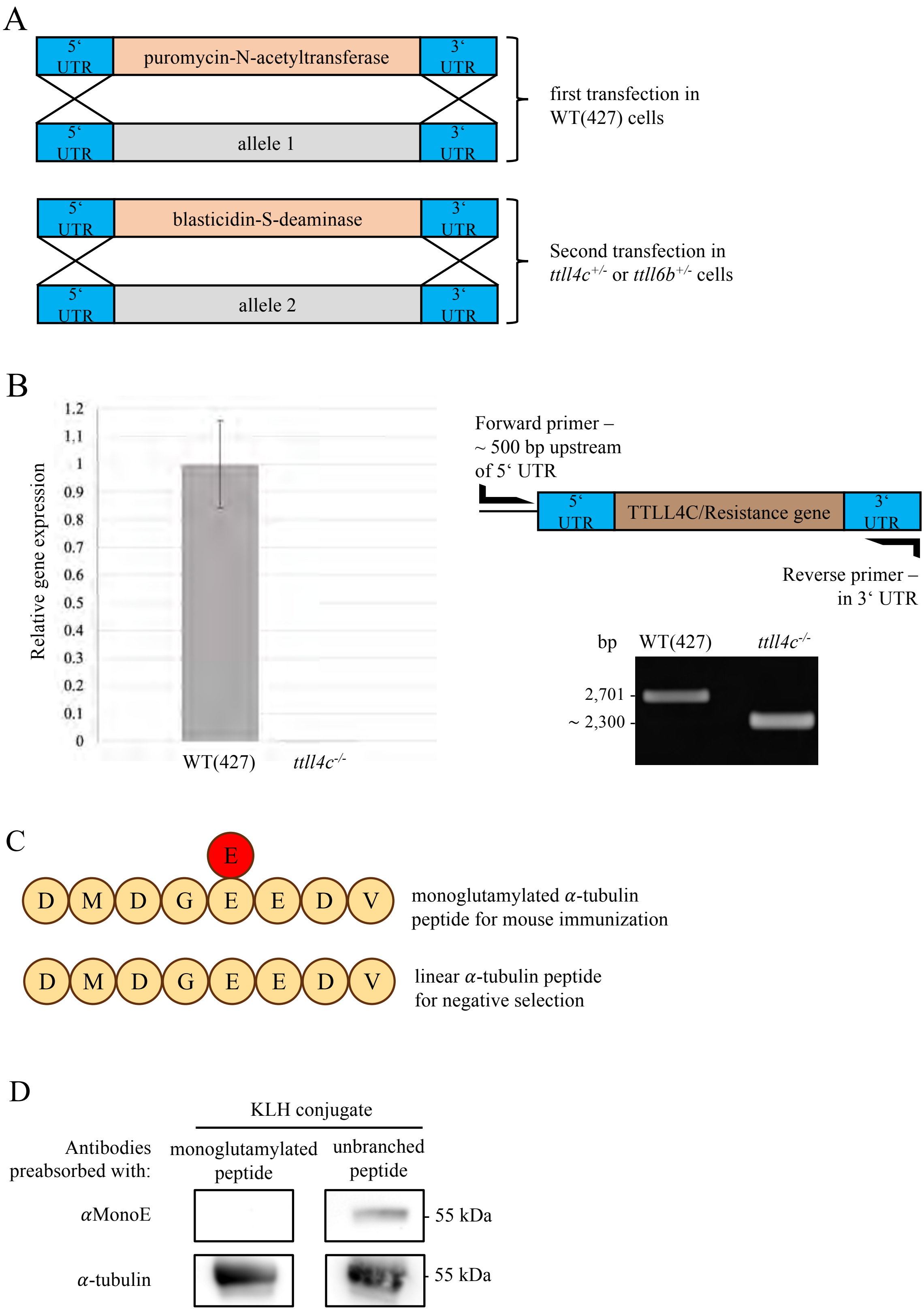

Supplementary Figure S1. Gene Knock-Out Strategy for TTLLs, Validation of the TTLL4C KO and αMonoE antibody. (A) Schematic of the allelic replacement via homologous recombination. (B) Left: RT-qPCR of WT and *ttll4c*$^{-/-}$ cell lines confirming the absence of TTLL4C mRNA expression in knockout cells. Right: Visualization of primer binding sites and the corresponding PCR of the genomic *TTLL4C* locus confirming the absence of the wild-type alleles in *ttll4c*$^{-/-}$ cells. (C) Amino acid sequences of the monoglutamylated peptide (branch in red) derived from the α-tubulin C-terminus and the linear α-tubulin C-terminus. (D) Western blot analysis for validating αMonoE antibody specificity. Trypanosomal cytoskeleton samples were blotted and incubated with antibodies αMonoE and TAT1 (α-tubulin). Prior to membrane incubation, each antibody was preabsorbed with either the monoglutamylated α-tubulin peptide or the unbranched peptide conjugated to mcKLH overnight.

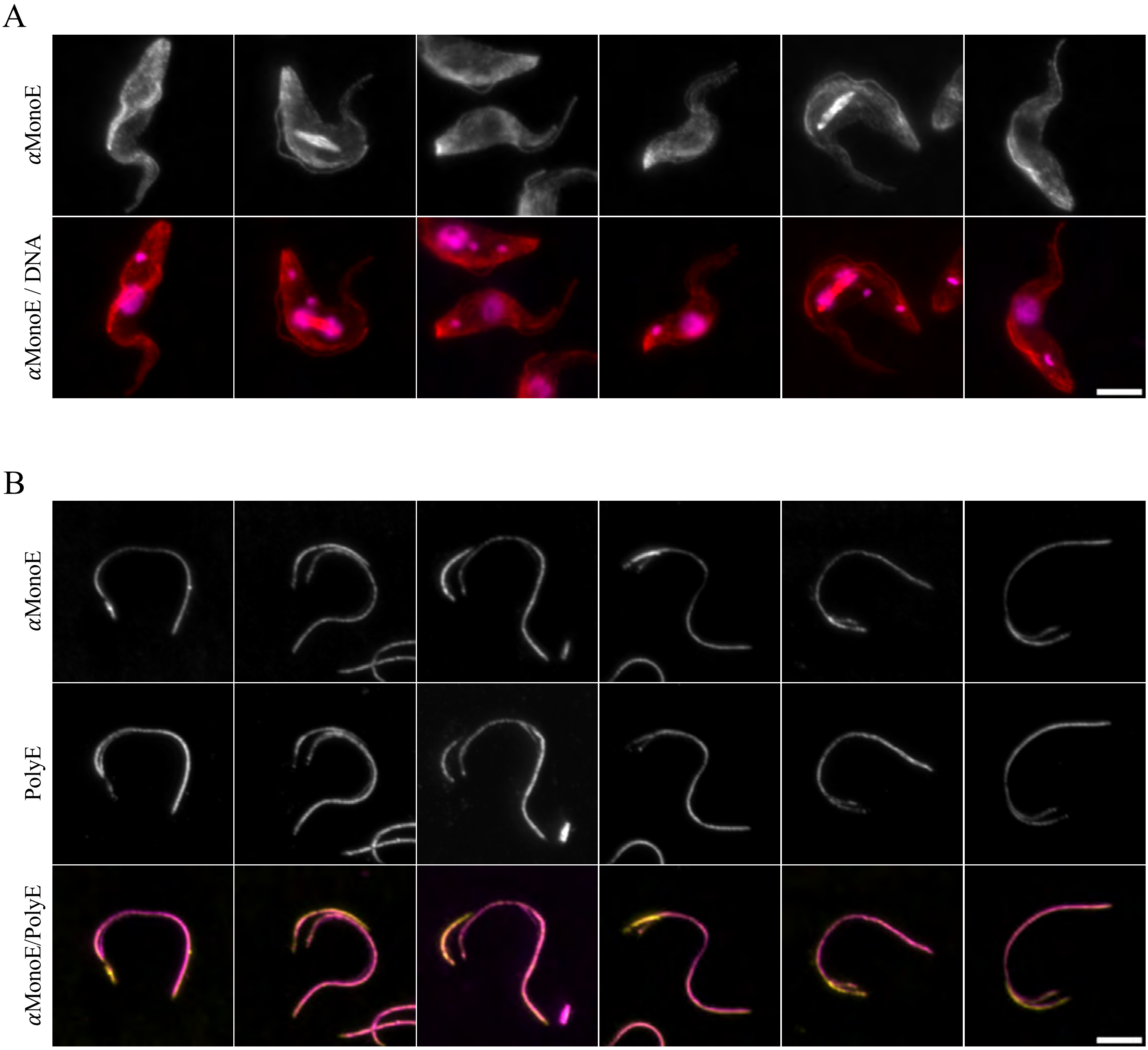

Supplementary Figure S2. Cell and flagella staining with the *α*MonoE antibody. (A) Immunofluorescence of WT(427) cells. Cells were stained with the *α*MonoE antibody (upper panels). Lower panels: *α*MonoE staining is colored in red. DNA was visualized with DAPI (magenta). (B) Flagellar preparations from WT(427) stained with *α*MonoE (upper panels) and PolyE (middle panels). The lower panels show the merge of both signals (*α*MonoE: yellow; PolyE: magenta). Scale bar: 5 µm.

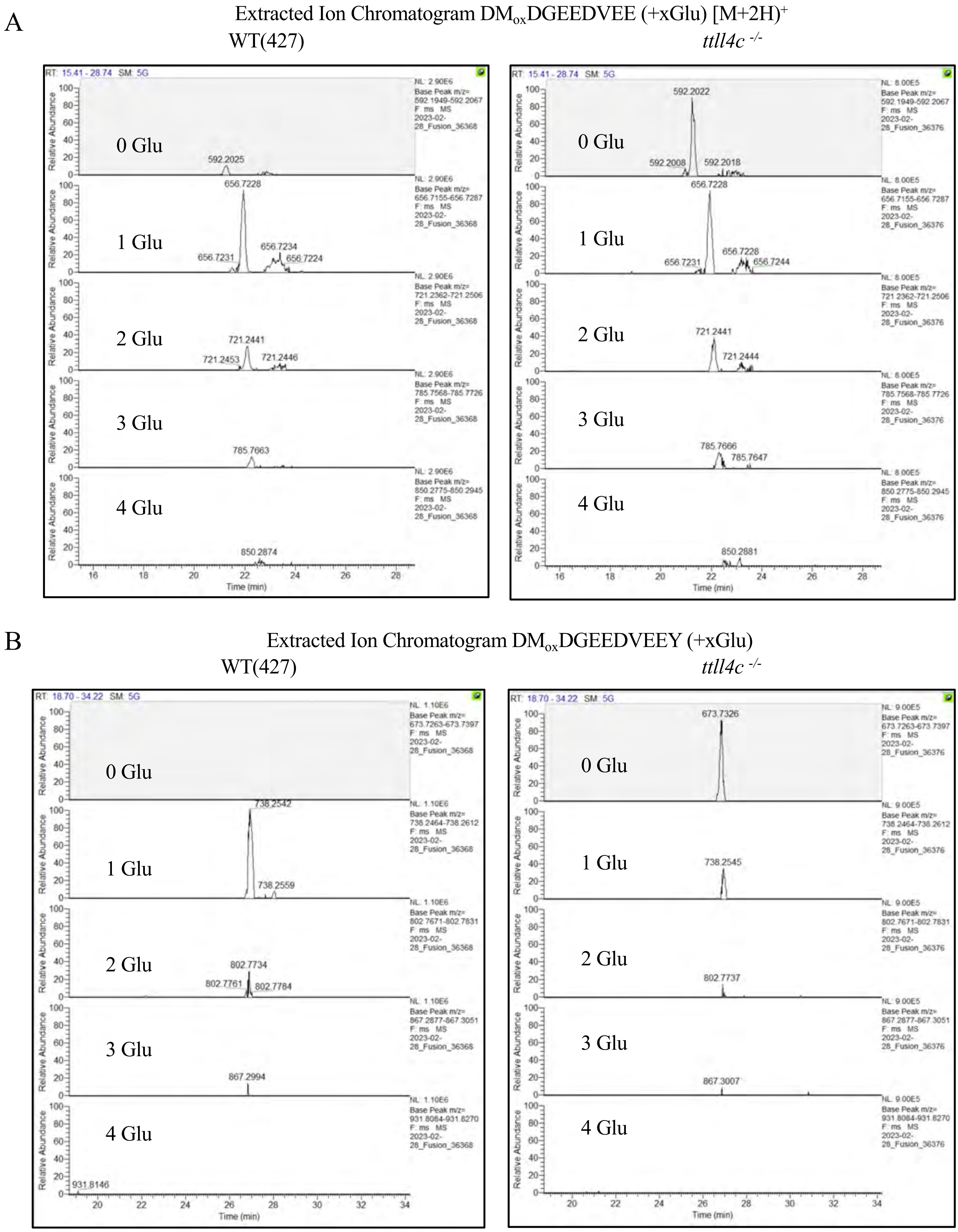

Supplementary Figure S3. TTLL4C Depletion Results in Unglutamylated α-Tubulin as Detected by Mass Spectrometry. (A) Extracted ion chromatogram of the detyrosinated C-terminal peptide of α-tubulin from WT and *ttll4c*$^{-/-}$ cells, showing glutamate chain lengths of 0–4 residues. Methionine oxidation is assumed. (B) Extracted ion chromatogram of the tyrosinated C-terminal peptide of α-tubulin from WT and *ttll4c*$^{-/-}$ cells, also showing glutamate chain lengths of 0–4 residues. Methionine oxidation is assumed.

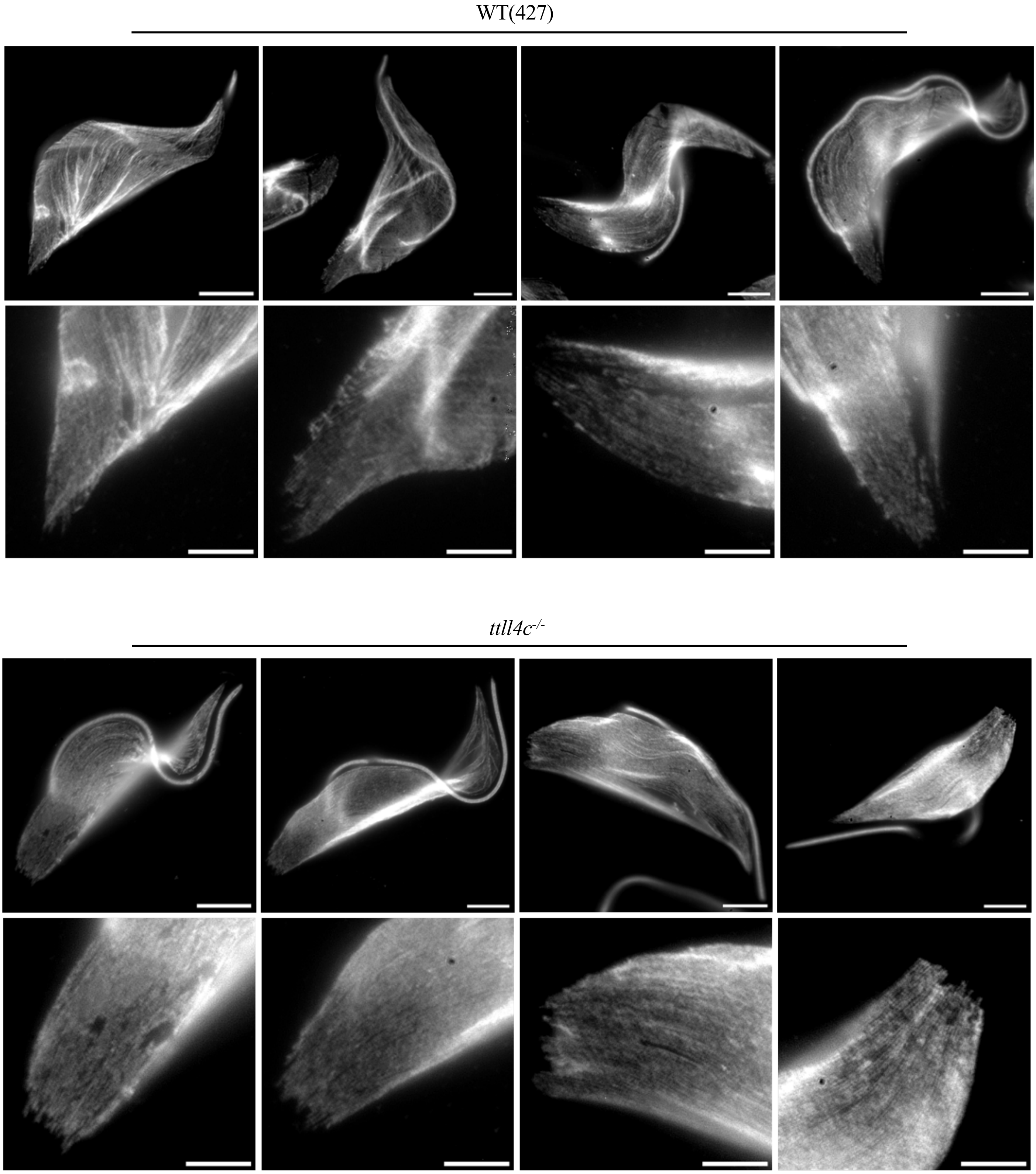

Supplementary Figure S4. Expansion microscopy of WT and *ttll4c*$^{-/-}$ cytoskeletons. Microtubules were stained with an α-tubulin antibody (TAT1). Upper panels show whole-cell views; lower panels display magnified images of the posterior tip. The images correspond to single z-slices. The magnified images (lower panels) were processed using the ImageJ tools "Enhance Contrast" and "Sharpen" using default settings. Scale bars for whole cell views: 10 µm. Scale bars for magnified posterior tips: 5 µm.

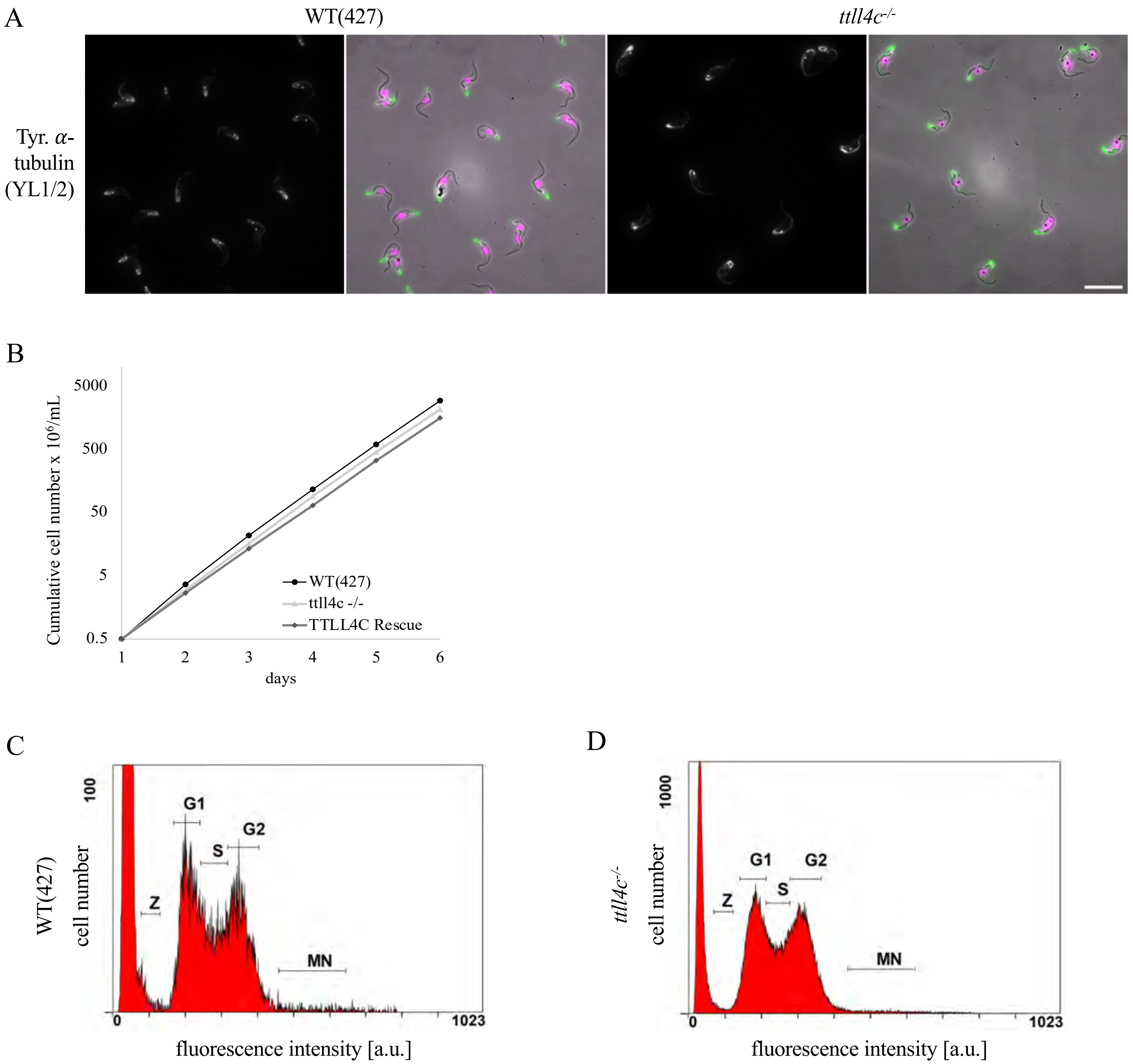

WT(427)	Z	G_1	S	G_2/M	MN	total
Proportion [%]	3.28	35.37	26.43	32.57	2.35	100

ttll4c$^{-/-}$	Z	G_1	S	G_2/M	MN	total
Proportion [%]	1.63	34.37	25.03	36.86	2.11	100

Supplementary Figure S5. Tyrosination, growth and cell cycle distribution of TTLL4C-deficient cells. (A) Immunofluorescence analysis of cytoskeletons from wild-type (WT) and *ttll4c*$^{-/-}$ cells. Tyrosinated α-tubulin was detected with the YL1/2 antibody (green), and DNA was stained with DAPI (magenta). The left panels show the YL1/2 signal alone, the right panels present merged fluorescence and phase-contrast images. Scale bar: 20 µm. (B) Logarithmic cumulative growth curves of the parental WT(427), *ttll4c*$^{-/-}$ and TTLL4C Rescue cell lines. Each data point represents the mean of three biological replicates, with each replicate measured in triplicate. (C-D) Flow cytometry profiles of propidium iodide stained cells. Z = zoids (cells lacking a nucleus); MN = multinucleate cells (cells carrying > 2 nuclei). The y-axis corresponds to the cell count of the respective cell lines. (C) Flow cytometry profile of WT(427) cells. (D) Flow cytometry profile of *ttll4c*$^{-/-}$ cells.

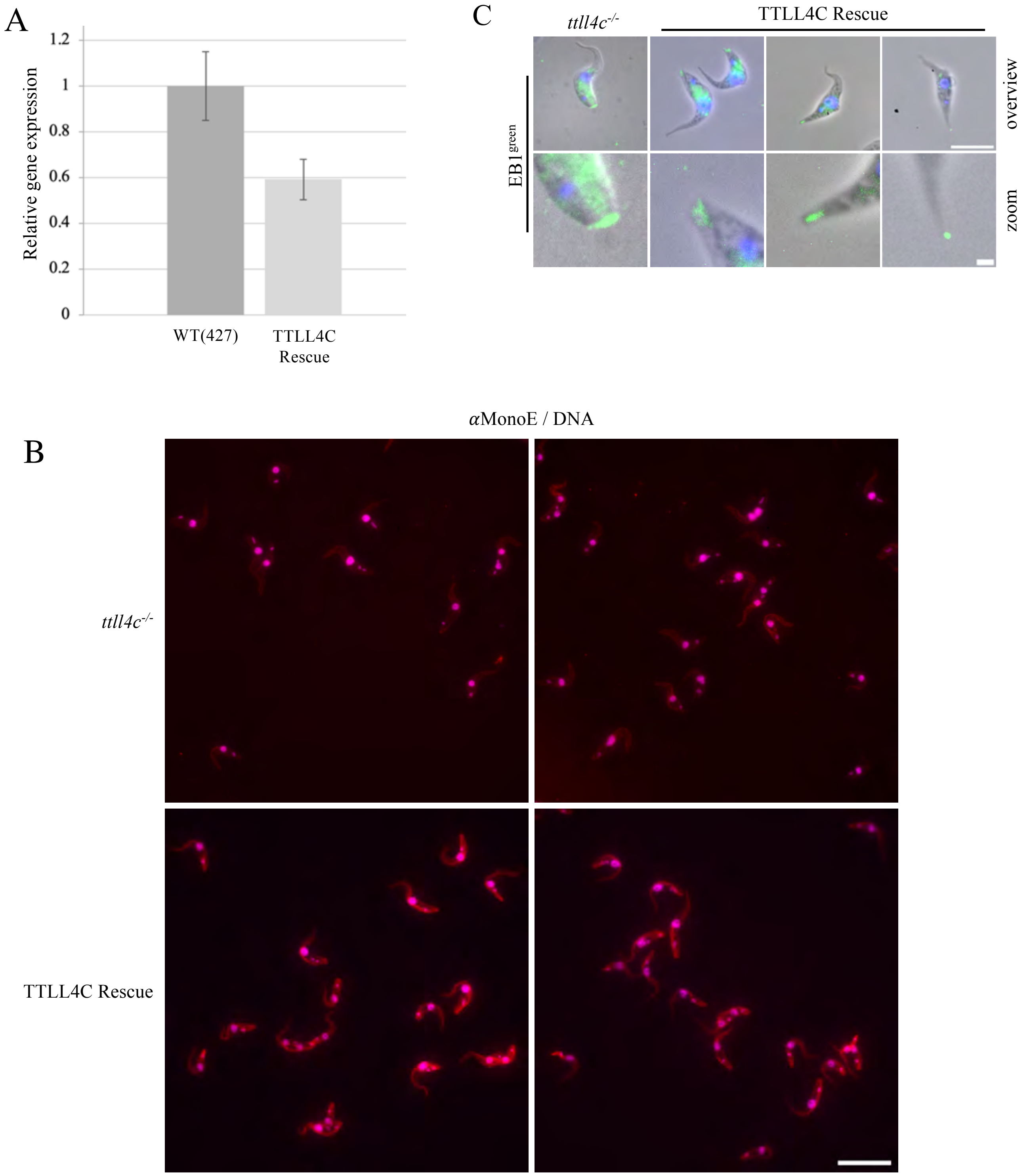

Supplementary Figure S6. TTLL4C expression level, αMonoE immunofluorescence and EB1 distribution in Rescue cells. (A) RT-qPCR comparing TTLL4C expression levels in the Rescue cell line (expressed from the tubulin locus via read-through transcription) to WT cells. (B) Immunofluorescence microscopy of *ttll4c*$^{-/-}$ and TTLL4C Rescue cytoskeletons. Cytoskeletons were labeled with the αMonoE (red) antibody. DNA (nucleus and kinetoplast) was stained using DAPI (magenta). Scale bar: 20 µm. (C) Immunofluorescence microscopy of *ttll4c*$^{-/-}$ and TTLL4C Rescue cells merged with differential interfence contrast (*ttll4c*$^{-/-}$) or phase contrast (TTLL4C Rescue) images . Whole cells labeled with anti-EB1 antibody (green) and DNA stained with DAPI (blue). Scale bars: 10 µm (whole cell view), 1 µm (posterior tips).

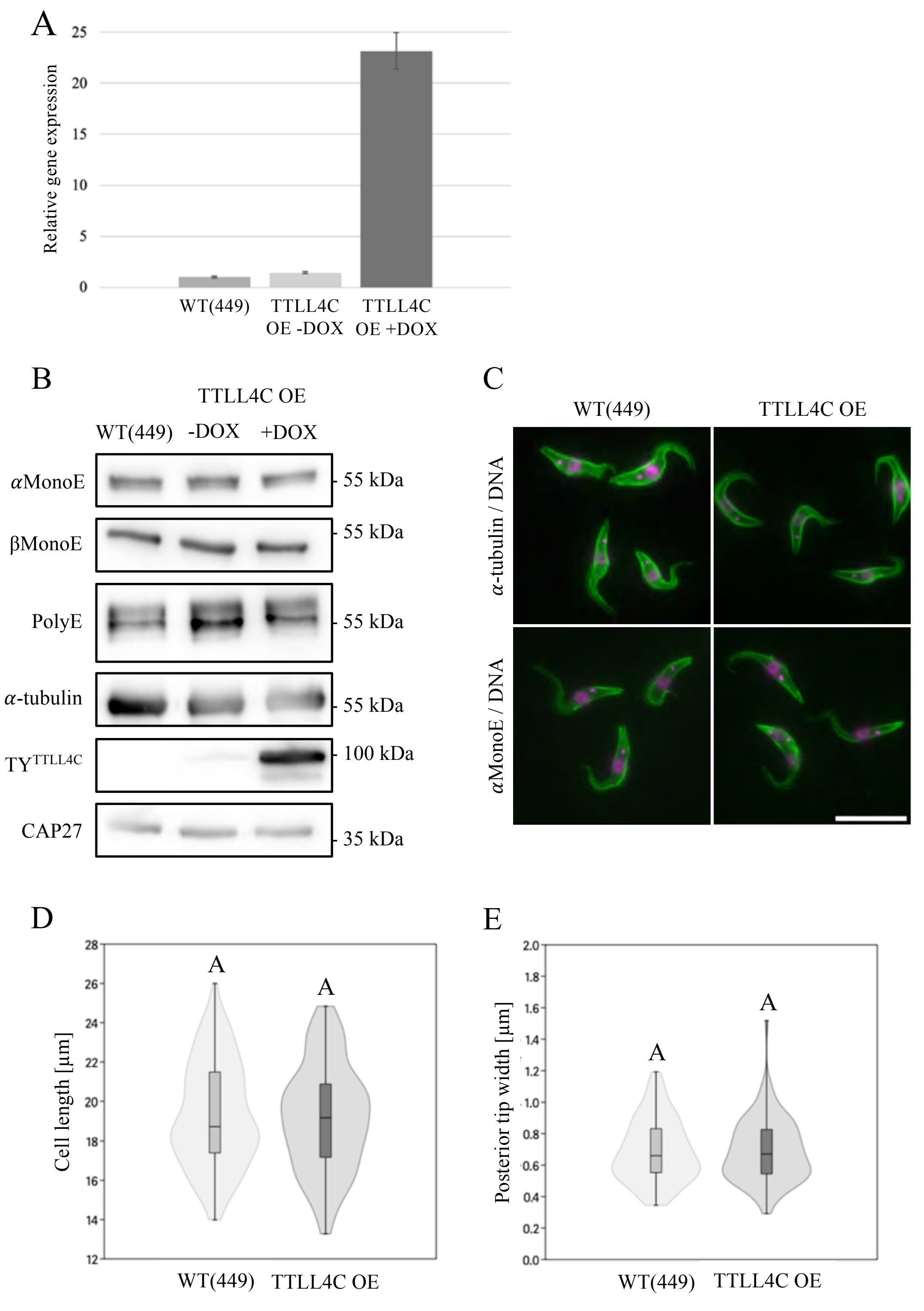

Supplementary Figure S7. Overexpression of TTLL4C. (A) RT-qPCR analysis of TTLL4C overexpression (OE) in cells with an inducible promoter (+DOX) inserted into the ribosomal spacer DNA, compared to both the parental WT(449) cell line and uninduced OE cells (-DOX). Error bars indicate standard error. (B) Western blot of cytoskeletons derived from WT(449) and 3xTy-TTLL4C overexpressing cells. α-tubulin (TAT1) and CAP27 serve as loading controls. (C) Immunofluorescence of WT(449) and 3xTy-TTLL4C overexpressing cells. Cells were stained with α-tubulin (TAT1, green; upper panels) and αMonoE (green; lower panels) antibodies. DNA was visualized with DAPI (magenta). Scale bar: 15 µm. (D) Violin plots depicting the distribution of cell lengths in WT(449) and TTLL4C overexpressing cells. (E) Analysis of posterior tip width distribution in G1-phase WT(449) and TTLL4C overexpressing cells. The plots display the arithmetic mean, interquartile range, standard error, and density curve of the data points. Sample size (N) = 100 cells. Statistical significance between groups is denoted by different letters, with identical letters indicating a lack of statistical significance (Mann-Whitney pairwise test, $p > 0.05$). The data presented are representative of three independent experiments. Ty-TTLL4C overexpression was induced for 3 days in all experiments.

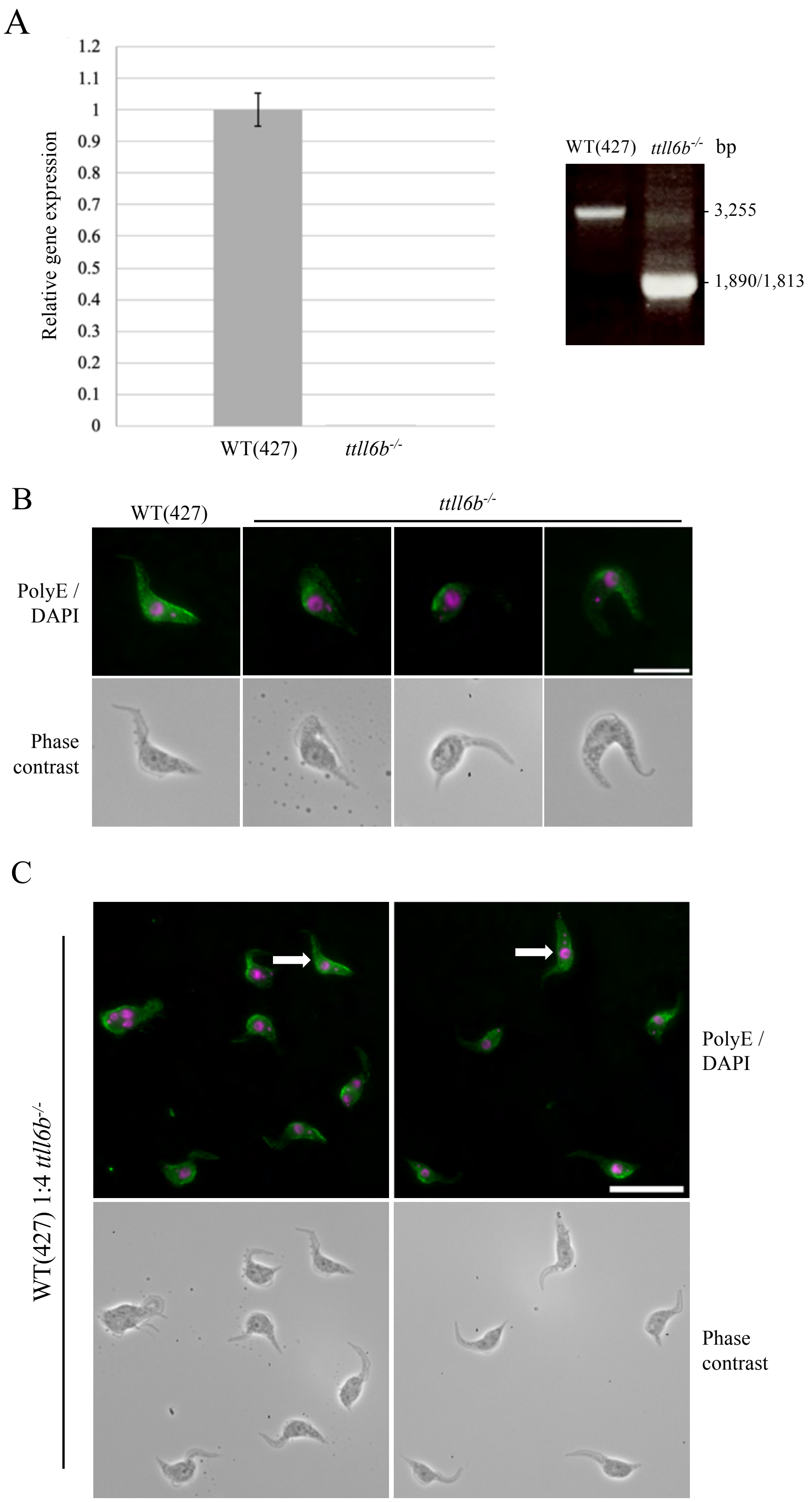

Supplementary Figure S8. qPCR and Microscopic Analysis of *ttll6b*$^{-/-}$ cells. (A) Left: RT-qPCR analysis of WT and *ttll6b*$^{-/-}$ cells confirming the absence of *TTLL6B* mRNA expression in knockout cells. Right: PCR of the genomic *TTLL6B* locus confirming the absence of the wild-type alleles in *ttll6b*$^{-/-}$ cells. (B) Immunofluorescence microscopy of WT and *ttll6b*$^{-/-}$ cells stained with PolyE (green; upper panels) and DNA labeled with DAPI (magenta). The lower panels display the corresponding phase contrast images. Scale bar: 10 µm. (C) Immunofluorescence microscopy of WT and *ttll6b*$^{-/-}$ cells mixed at a 1:4 ratio, stained with PolyE and DAPI (upper panels). The lower panels show the corresponding phase contrast images. Wild-type cells are marked with arrows. Images are representative of three independent experiments. Scale bar: 20 µm.

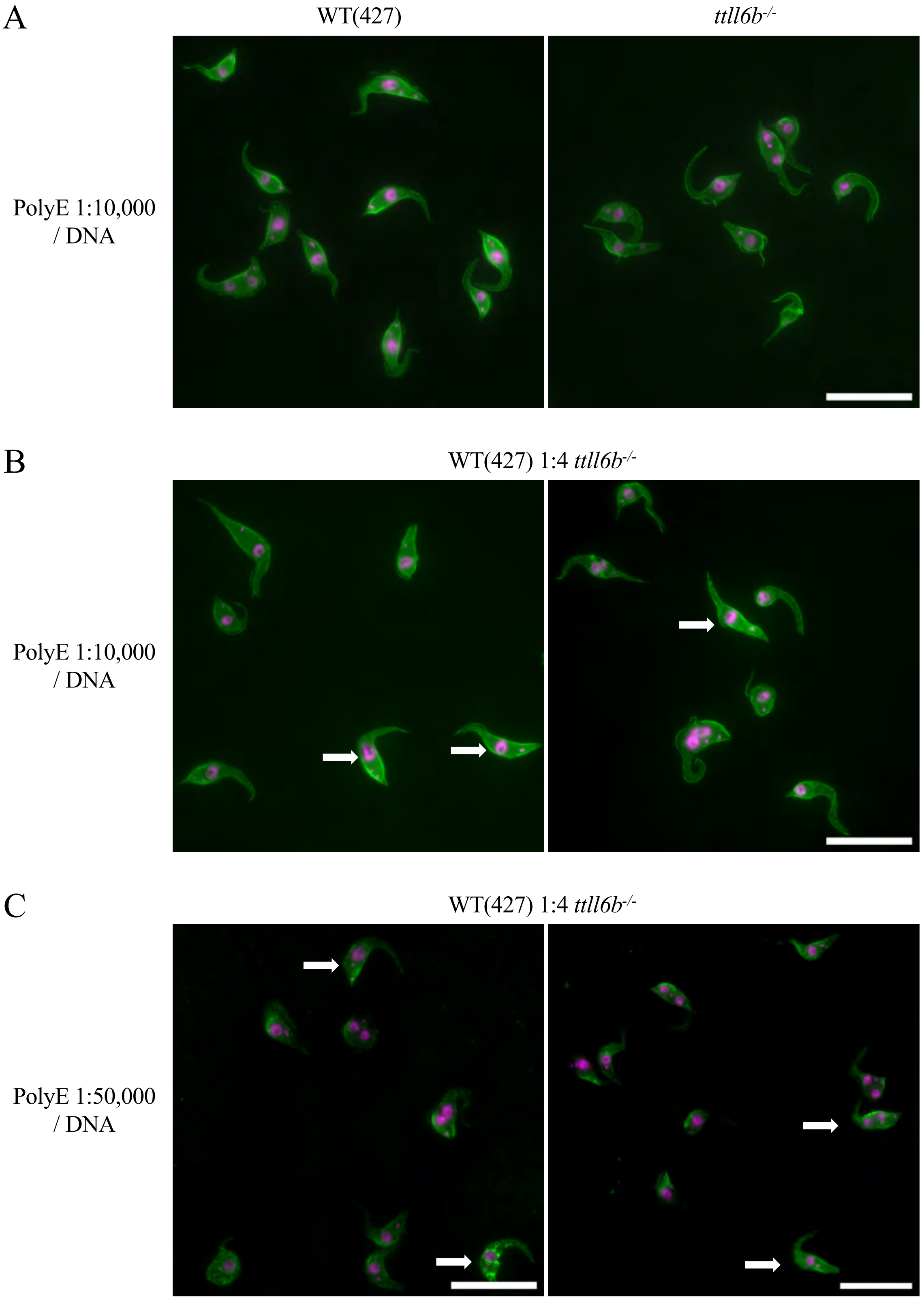

Supplementary Figure S9. PolyE dilutions for WT(427) and *ttll6b*$^{-/-}$ discrimination. (A) Immunofluorescence microscopy of WT and *ttll6b*$^{-/-}$ cells stained with PolyE (1:10,000; green) and DNA labeled with DAPI (magenta). (B) Immunofluorescence microscopy of WT and *ttll6b*$^{-/-}$ cells mixed at a 1:4 ratio, stained with PolyE (1:10,000) and DAPI. (C) Immunofluorescence microscopy of WT and *ttll6b*$^{-/-}$ cells mixed at a 1:4 ratio, stained with PolyE (1:50,000) and DAPI. Brighter, wild-type cells are marked with arrows. Scale bar: 20 µm.

A

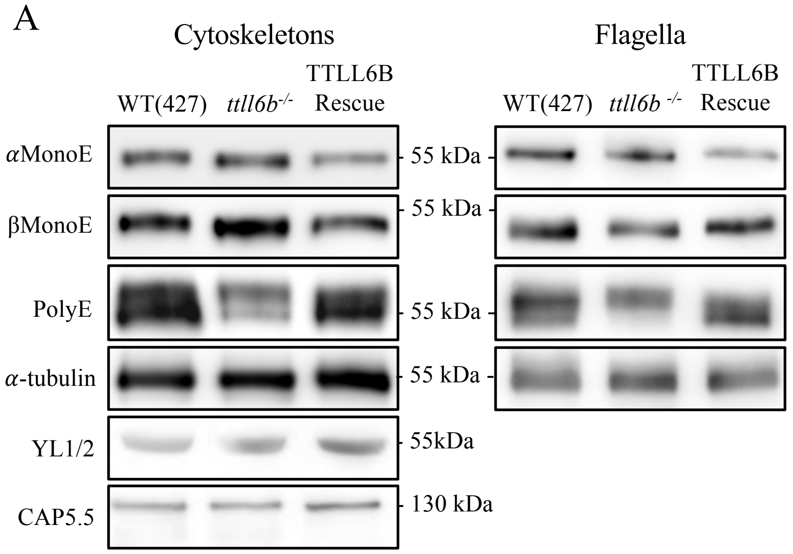

B

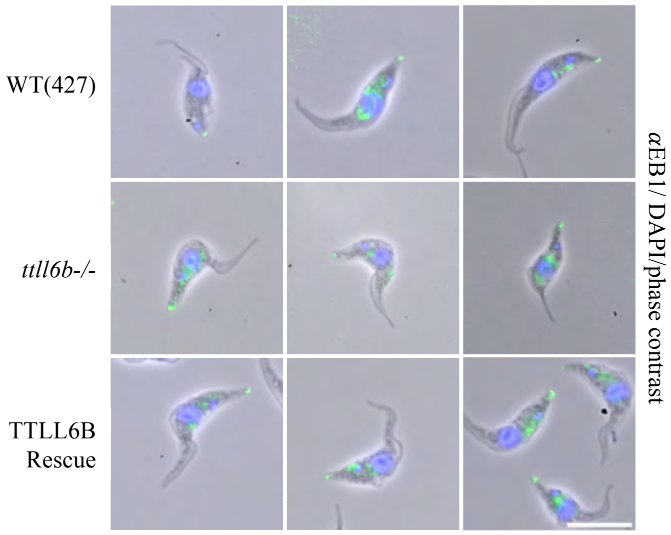

C

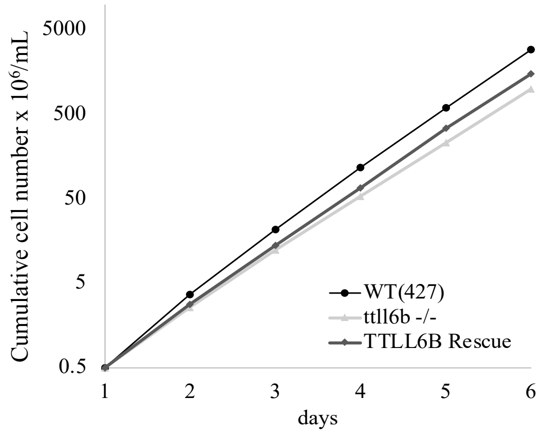

D

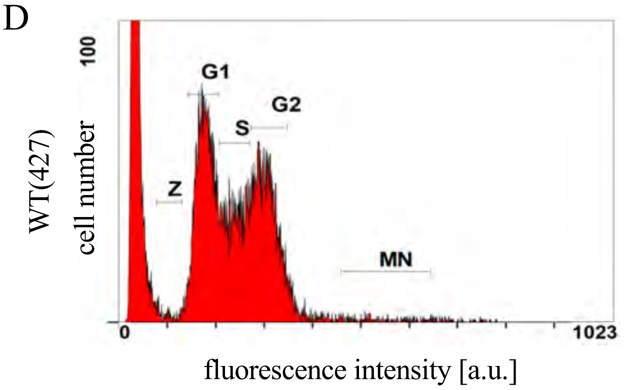

WT(427)	Z	G_1	S	G_2/M	MN	total
Proportion [%]	1.47	37.14	25.85	33.80	1.75	100

E

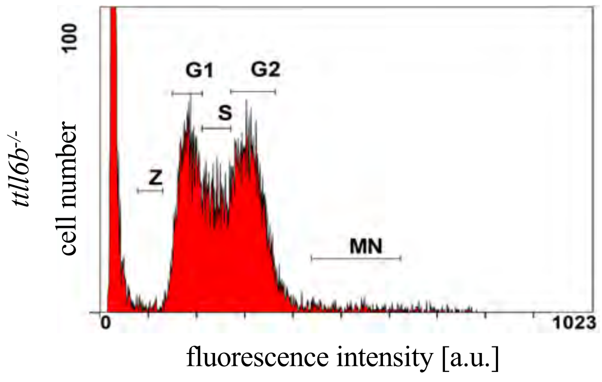

ttll6b$^{-/-}$	Z	G_1	S	G_2/M	MN	total
Proportion [%]	1.02	30.92	24.10	41.32	2.64	100

F

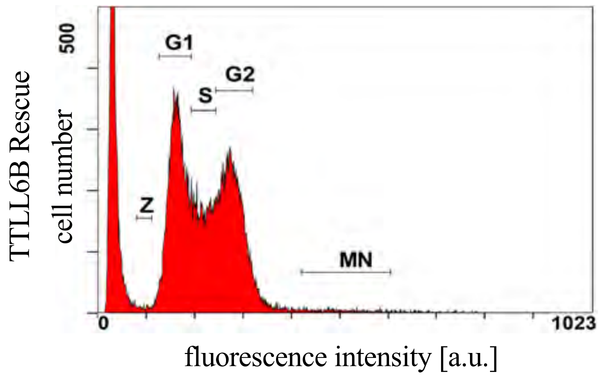

TTLL6B Rescue	Z	G_1	S	G_2/M	MN	total
Proportion [%]	0.57	37.46	24.74	35.28	1.94	100

Supplementary Figure S10. Biochemical, morphological and growth analysis of TTLL6B cell lines. (A) Western blots of cytoskeletal and flagellar fractions of WT, *ttll6b*$^{-/-}$ and TTLL6B Rescue cells. (B) Immunofluorescence microscopy of WT, *ttll6b*$^{-/-}$ and TTLL6B Rescue cells. Whole cells labeled with anti-EB1 antibody (green) and DNA stained with DAPI (blue). The panels represent merged fluorescence and phase-contrast images. Scale bar: 10 µm. Images are representative of three independent experiments. (C) Logarithmic cumulative growth curves of the parental WT(427), *ttll6b*$^{-/-}$ and TTLL6B Rescue cell lines. Each data point represents the mean of three biological replicates, with each replicate measured in triplicate. (D-F) Flow cytometry profiles of propidium iodide stained cells (Z = zoids; MN = multinucleate cells). The y-axis corresponds to the cell count of the respective cell lines. (D) Flow cytometry profile of WT(427) cells. (E) Flow cytometry profile of *ttll6b*$^{-/-}$ cells. (F) Flow cytometry profile of TTLL6B Rescue cells.

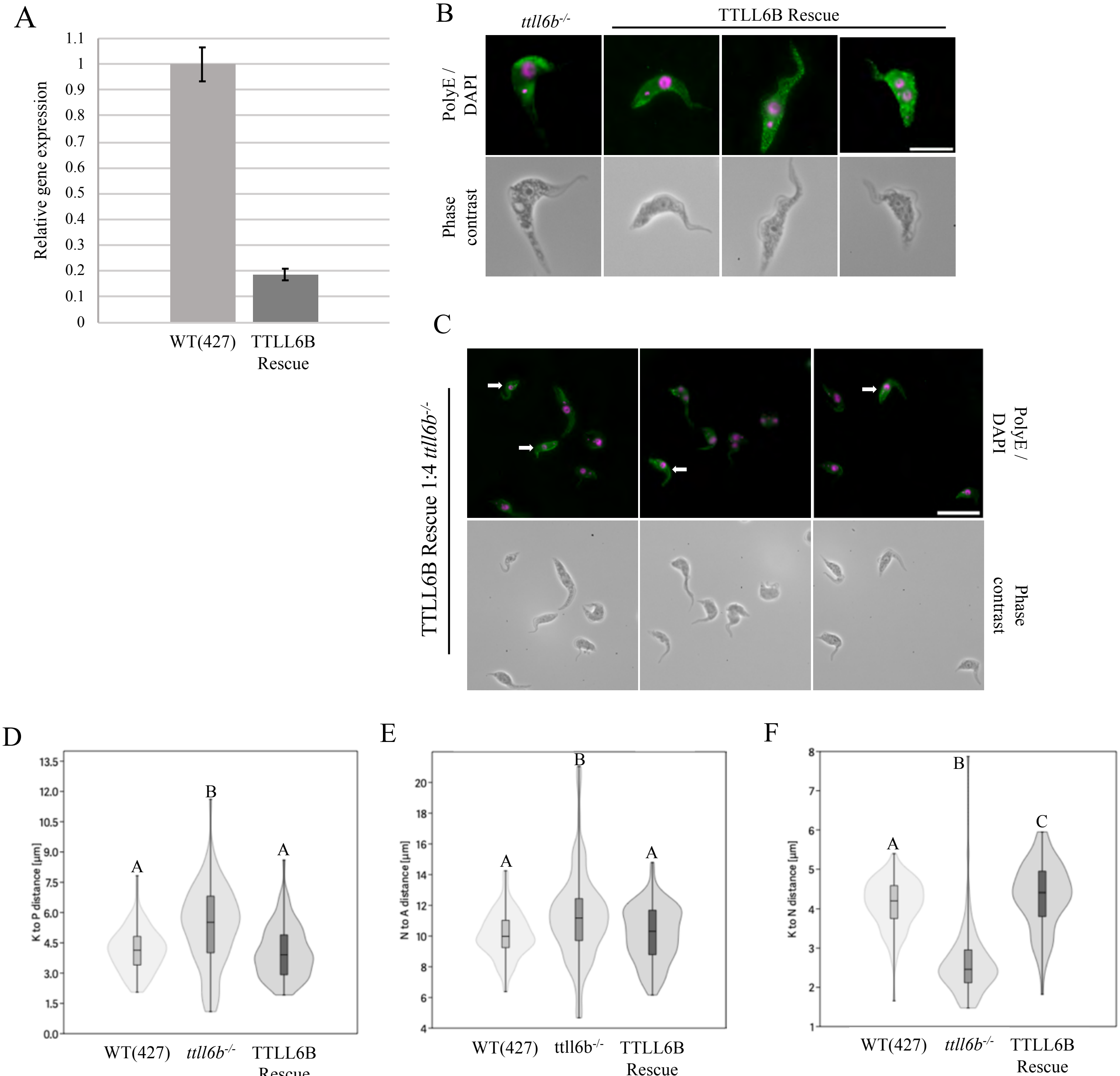

Supplementary Figure S11. Phenotypic Rescue by Ectopic Expression of TTLL6B. (A) RT-qPCR comparing TTLL6B expression in the Rescue cell line (transcription from the tubulin locus) to that in WT cells. Error bars indicate standard error. (B) Immunofluorescence microscopy of TTLL6B Rescue and *ttll6b*$^{-/-}$ cells. Rescue and mutant cells were stained with PolyE (green) antibodies, while DNA (nucleus and kinetoplast) was stained with DAPI (magenta). Images are representative of three independent experiments. The panels represent merged fluorescence and phase-contrast images. Scale bar: 10 µm. (C) Immunofluorescence microscopy of TTLL6B Rescue and *ttll6b*$^{-/-}$ cells. Rescue and mutant cells were mixed at a 1:4 ratio and stained with PolyE (green) antibody, while DNA (nucleus and kinetoplast) was stained with DAPI (magenta). The panels represent merged fluorescence and phase-contrast images. Rescue cells are marked with arrows. Scale bar: 20 µm. All images are representative of three independent experiments. (D) Violin plots illustrating the distribution of the kinetoplast-to-posterior tip (K-P) length in WT, *ttll6b*$^{-/-}$ mutant and TTLL6B Rescue cells. (E) Distribution of nucleus-to-anterior tip (N-A) lengths in G1-phase WT, *ttll6b*$^{-/-}$ and TTLL6B Rescue cells. (F) Analysis of the kinetoplast-to-nucleus (K-N) distance in G1-phase WT, *ttll6b*$^{-/-}$ and TTLL6B Rescue cells. The violin plots depict the arithmetic mean, interquartile range, standard error, and density curves of the measurements. Sample size (N) = 100 cells. Statistical significance between groups is indicated by different letters, with identical letters denoting no statistical significance (Mann-Whitney pairwise test, $p < 0.05$). Data shown are representative of three independent experiments.

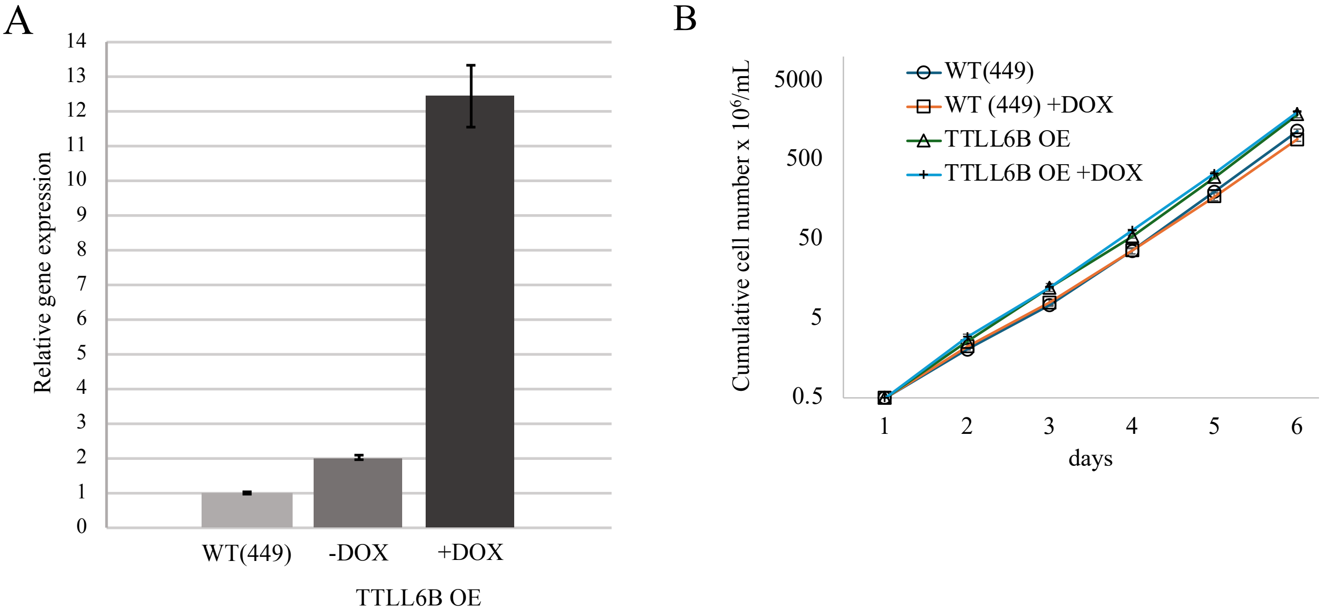

Supplementary Figure S12. Growth Curves and Cell Cycle Progression of TTLL6B Cell Lines. (A) RT-qPCR of TTLL6B overexpression (OE) in cells with an inducible promoter (+DOX) inserted into ribosomal spacer DNA, compared to the parental WT(449) cell line and uninduced OE cells (-DOX). (B) Logarithmic cumulative growth curves of the parental WT(449) [grown with (+) or without doxycycline (DOX)] and TTLL6B-overexpressing (OE) cell lines under both +DOX and −DOX conditions. Each data point represents the mean of three biological replicates, with each replicate measured in triplicate.
